# Virulome over taxonomy: refining the driver-passenger model in colorectal carcinogenesis

**DOI:** 10.3389/fcimb.2026.1858868

**Published:** 2026-07-14

**Authors:** Evgeniya Glazunova, Alexander Kurnosov, Anastasia Bogacheva, Valentin Makarov, Olga Zlobovskaya

**Affiliations:** 1 Federal State Budgetary Institution (Centre for Strategic Planning and Management of Biomedical Health Risks) of the Federal Medical Biological Agency, Moscow, Russia; 2 Emanuel Institute of Biochemical Physics of Russian Academy of Sciences, Moscow, Russia

**Keywords:** biomarkers, carcinogenesis, colorectal cancer, colorectal cancer hypotheses, CRC diagnosis, functional profiling, human gut microbiota, polymicrobial interactions

## Abstract

Colorectal carcinogenesis is increasingly viewed as a predominantly microbiome-driven process, yet it extends well beyond simple taxonomic associations. The taxon-based classical driver-passenger model, while conceptually useful, may benefit from incorporating the underlying mechanisms of microbial participation and the functional complexity of taxa contributions across CRC stages. In particular, the same taxa may exert distinct effects across carcinogenesis, and individual metabolic pathways frequently mediate multiple and divergent host responses. To address these limitations, we re-examine the roles of CRC-associated microbiota through the lens of individual virulence factor effects on the host, demonstrating their functional pleiotropy, and propose an expanded driver-passenger model. This approach highlights the central role of the virulome in CRC initiation, promotion and progression. By shifting the analytical focus from taxonomy to function, the proposed framework enables improved causality assessment and supports the development of stage-specific diagnostic and prognostic markers, as well as more targeted microbiome-directed therapeutic strategies.

## Introduction

Recent years have seen sporadic colorectal cancer (CRC) research expand into several major directions, including host genetic susceptibility (Clay et al., 2022; Pleguezuelos-Manzano et al., 2022; Dougherty and Jobin, 2023; Hu et al., 2025) and tumor mutational landscape (Pleguezuelos-Manzano et al., 2020; Hu et al., 2025; Shi et al., 2025), the cancer-associated metabolome (Yachida et al., 2019; Clos-Garcia et al., 2020; Han, 2025), and the tumor microenvironment (TME) (Glazunova et al., 2025; Bautista et al., 2026), particularly its crosstalk with the microbiota. In parallel, increasing attention has been paid to intratumoral microbial heterogeneity; microbial profiling (“carcinogenic enterotypes”) (Zwinsová et al., 2021; Mouradov et al., 2023; Han, 2025; Hu et al., 2025; Karaman et al., 2025); host-bacterial interactions mediated by virulence factors (He et al., 2019; Gaab et al., 2023; Tozzi et al., 2025; Xia et al., 2025); the emerging roles of the archaeome (Glazunova et al., 2025), mycobiome (Pleguezuelos-Manzano et al., 2022), and transient infections (including parasitic infections as active carcinogenic contributors) (Glazunova et al., 2025), as well as to their cumulative impact on colorectal carcinogenesis (Cheng et al., 2020; Glazunova et al., 2025).One of the most consistently documented observations in sporadic CRC research is the association between gut microbial imbalance and disease development (Nagtegaal et al., 2019; Yachida et al., 2019; Cheng et al., 2020; Villar-Ortega et al., 2022; Glazunova et al., 2025; Bautista et al., 2026). Numerous studies have highlighted how opportunistic and pathogenic microorganisms drive colorectal carcinogenesis through their virulence factors. Among these, the “big trio” stands out: *Fusobacterium nucleatum* (over 10, 000 PubMed entries linking this bacterium to CRC since 1960, with a record 2, 256 publications in 2025 alone), pks-positive *Escherichia coli* (3, 244 PubMed records since 1959, peaking at 579 publications in 2025), and Enterotoxigenic subtype of *Bacteroides fragilis* (ETBF; 3, 196 PubMed entries since 1978, reaching 504 publications in 2025) (Boleij et al., 2015; Drewes et al., 2017; Dejea et al., 2018; Clay et al., 2022; Villar-Ortega et al., 2022; Dougherty and Jobin, 2023; Gaab et al., 2023; Incognito et al., 2025; Bautista et al., 2026), and, in the case of ETBF, with an increased risk of metastasis (Bullman et al., 2017; Saito et al., 2025). Of particular note, investigations into the association between ETBF and CRC led Sears and Pardoll to formulate the “Alpha-bug” hypothesis in 2011 (Sears and Pardoll, 2011), suggesting that infection with ETBF directly triggers inflammation and subsequent carcinogenesis.

Beyond these three key taxa, researchers are actively exploring the roles of *Peptostreptococcus anaerobius* (Long et al., 2019; Liu et al., 2024; Piccinno et al., 2025), *Parvimonas micra* (Purcell et al., 2017; Yachida et al., 2019; Glazunova et al., 2025; Piccinno et al., 2025), *Enterococcus faecalis* (Golińska, 2013; Ferchichi et al., 2021), other *Fusobacterium* species (Purcell et al., 2017; Lawrence et al., 2020; Zepeda-Rivera et al., 2024; Piccinno et al., 2025), *Streptococcus* spp. (particularly *S. gallolyticus* subsp. *gallolyticus*) (Aymeric et al., 2018; Harrington et al., 2021), *Campylobacter* spp (Gemmell et al., 2018; Liu et al., 2018; He et al., 2024)., *Porphyromonas* spp (Purcell et al., 2017; Mu et al., 2020)., *Prevotella* spp., and the family *Enterobacteriaceae* (Glazunova et al., 2025) in gastrointestinal inflammation and tumorigenesis (Cheng et al., 2020).

Various studies have shown that taxa with tumor-initiating activity and subsequent tumor-enriched taxa display distinct patterns of enrichment across CRC stages, with statistically significant stage-specific differences. The classic tumor-associated *P. micra* is predominantly elevated in advanced CRC (AUROC, Area Under the Receiver Operating Characteristic 0.81 (Clos-Garcia et al., 2020); reaching AUROC 0.91 for later stage with distant metastasis (Osman et al., 2021)). Notably, it also discriminated adenoma from controls with AUROC 0.73 (0.66-0.80), challenging its strict status (Wong et al., 2017). *P. anaerobius* performs somewhat less convincingly in the same reports (adenoma vs. control - AUROC 0.72 (0.65-0.80) (Wong et al., 2017); advanced stages vs. control - AUROC 0.76 (Clos-Garcia et al., 2020)). Numerous studies show that *F. nucleatum* group (sensu lato) is more robustly associated with advanced CRC (AUROC 0.71 (Löwenmark et al., 2020); 0.875 (Guo et al., 2018); 0.87 (Clos-Garcia et al., 2020); 0.83 (0.78-0.89) (Wong et al., 2017); 0.868 (Liang et al., 2017)) than with adenoma or early CRC (0.59 (0.51-0.67) (Wong et al., 2017); 0.52 (Xie et al., 2017); 0.635 (Guo et al., 2018)). These findings suggest the *F. nucleatum* group predominantly acts as a progression-associated tumor colonizer.

*Escherichia* and *Bacteroides* species demonstrate diagnostic utility only in combination with other key markers for CRC detection. For these genera, single-taxon AUROC values reached 0.71 (Deng et al., 2025); while a multi-taxon panel including *F. nucleatum* sensu stricto, *F. varium*, *F. hwasookii*, *P. gingivalis*, *P. micra*, *P. asaccharolytica*, *P. intermedia*, *E. coli*, *Citrobacter freundii*, and *B. fragilis* showed AUROC 0.800 (Gao et al., 2021).

Combining taxa into panels also improves overall diagnostic discrimination between early-stage and advanced tumor lesions (Wu et al., 2023). The combination *P. stomatis* + *P. micra* + ETBF, for example, discriminated Laterally Spreading Tumor (LST) from Colorectal Adenoma (AUROC 0.84) and LST from healthy controls (AUROC 0.92) (Shen et al., 2021). In another cohort dominated by advanced metastatic CRC, only four taxa (*P. micra*, *P. stomatis*, *F. nucleatum* group, *A. muciniphila*) were sufficient to distinguish patients from healthy controls with high accuracy (AUROC 0.927) (Osman et al., 2021).

In their seminal work, Tjalsma and colleagues (Tjalsma et al., 2012) proposed viewing colorectal carcinogenesis as the outcome of complex, evolving interspecies interactions, and introduced the driver-passenger model. These interactions lead to a shift in the intestinal microbial community profile from oncogenic initiators (“drivers”) to bacterial passengers that partially outcompete the drivers. This concept has since become a foundational framework in CRC-related microbiome research (Raskov et al., 2018; Wirbel et al., 2019; Cheng et al., 2020; Avril and DePaolo, 2021; Hua et al., 2022; Mathlouthi et al., 2022; Xing et al., 2022; Incognito et al., 2025; Bautista et al., 2026). It has informed numerous studies (Wirbel et al., 2019; Glazunova et al., 2025) and is supported by extensive experimental evidence elucidating the underlying biological mechanisms of CRC initiation (Li et al., 2021; Clay et al., 2022; Mathlouthi et al., 2022), progression (Clay et al., 2022; Mathlouthi et al., 2022; Zhang et al., 2022; Dougherty and Jobin, 2023; Gaab et al., 2023), and clinical outcomes (Purcell et al., 2017; Younginger et al., 2023; Incognito et al., 2025; Xia et al., 2025; Bautista et al., 2026).

Over the past decade, the research community’s focus has broadened from studying the functional roles of individual microorganisms, their toxic metabolites, and virulence factors (Yachida et al., 2019; Cheng et al., 2020; Avril and DePaolo, 2021; Mathlouthi et al., 2022; Dougherty and Jobin, 2023; Incognito et al., 2025; Bautista et al., 2026) toward understanding the stepwise, synergistic participation of structured carcinogenic communities (Li et al., 2021; Bautista et al., 2026; Bautista et al., 2026). This includes their metabolic networks, quorum-sensing communication (Ferchichi et al., 2021; Harrington et al., 2021), and spatial organization within biofilms (Raskov et al., 2018; Cheng et al., 2020; Avril and DePaolo, 2021; Clay et al., 2022; Xing et al., 2022; Incognito et al., 2025; Bautista et al., 2026), along with the various pro-oncogenic mechanisms: barrier disruption (Li et al., 2021; Bautista et al., 2026) inflammation induction (Wu et al., 2009; Arthur et al., 2012), genotoxicity, cytotoxicity (Chung et al., 2018; He et al., 2019; Cheng et al., 2020), and immune modulation (Gur et al., 2015; Chung et al., 2018).

Taken together, it is increasingly evident that the terms drivers and passengers (Hajishengallis et al., 2012) refer not simply to specific keystone virulence species (Alexander et al., 2018; Scott et al., 2019). Rather, they represent broader conceptual entities encompassing microorganisms, their virulence profiles, metabolites, and ultimately collective functional roles associated with particular stages of tumor progression (Scott et al., 2019; Clos-Garcia et al., 2020; Wang et al., 2020; Mathlouthi et al., 2022; Piccinno et al., 2025). Another key point is that most publications focus on associative rather than causal links for such effects and thus, only partially characterize the intratumoral evolution of the CRC-related microbiome (Alexander et al., 2018; Piccinno et al., 2025). Distinguishing active disease contributors from bystander taxa across the entire colorectal carcinogenesis continuum is challenging (Scott et al., 2019). Profiling of virulence factors helps identify microbes actively contributing to CRC.

In this review, we examine the CRC-associated microbial consortium through the dynamic framework of the driver-passenger hypothesis (Tjalsma et al., 2012), considering both the taxa involved and the specific virulence factors they harbor. We summarize evidence on the key virulence factors and functional roles of microbial communities from early carcinogenic initiators to late-stage passengers. To capture the non-binary nature of microbial involvement, we propose distinguishing primary (alpha) drivers, “second-hit” drivers, and active late-stage colonizers, and discuss how specific taxa switch roles across CRC progression based on deployed virulence functions.

## CRC-associated microbiota: taxonomy and etiology

Among taxa with the strongest evidence for CRC association, a substantial proportion are typical oral bacteria (Drewes et al., 2017; Kudra et al., 2023; Qin et al., 2024; Piccinno et al., 2025) ranging from opportunists (*Fusobacterium* spp., *Treponema* spp., *Selenomonas* spp., *P*. *intermedia*, *P. micra*) and pathogens at higher loads (*P*. *gingivalis*) to commensals and beneficial symbionts (*Streptococcus* spp., *Prevotella* spp., *Leptotrichia* spp., *Actinomyces* spp., *Gemella morbillorum*). Among fungi, *Candida albicans* deserves particular mention.

Commensal and opportunistic gut taxa harboring CRC and IBD associated virulence determinants include, beyond the big trio species, members of *Enterobacteriaceae* (*Salmonella* spp., *Shigella* spp., *Citrobacter* spp., *Cronobacter* spp., *Klebsiella* spp., *Enterobacter* spp.), *Morganellaceae*, and genera *Streptococcus*, *Bacteroides*, *Campylobacter*, *Peptostreptococcus*, *Enterococcus*, and *A. actinomycetemcomitans*. These taxa are predominantly anaerobes and proteolytic bacteria linked to dysbiosis and inflammation.

In the context of carcinogenesis, dysbiosis is defined as a persistent alteration in microbiome composition and function from a homeostatic state, characterized by reduced microbial diversity, depletion of beneficial commensals, and enrichment of pathobionts (Paduraru et al., 2025; Bautista et al., 2026). However, no universally accepted quantitative threshold for a “normal” microbiome exists, owing to substantial inter-individual variation. Accordingly, dysbiosis is increasingly viewed not as the presence of isolated pathogens but as an ecological shift in which the microbial community collectively facilitates tumor initiation and/or sustains tumor growth (Scott et al., 2019).

However, the gut microbiome is represented not only by taxa associated with CRC initiation and progression, but also by species involved in maintaining systemic homeostasis and intestinal barrier integrity, as well as performing several other functions (such as modulating metabolism and inflammatory responses) (Ionescu et al., 2025). Moreover, gut microbiota produce major short-chain fatty acids that nourish colonocytes, reinforce the epithelial barrier, and attenuate inflammation (Strain et al., 2022; Glazunova et al., 2025; Tingler et al., 2026), as well as antimicrobial peptides, amino acids, and vitamins (Chen et al., 2024; Garvey, 2024). Given the complex, dual role of the gut microbial community, the pathogenesis of CRC represents a dysfunction and reorganization of the gut microbial ecosystem (Ascandari et al., 2026).

## Driver-passenger hypothesis: evolution and perspective

The driver-passenger hypothesis (Tjalsma et al., 2012) reframes CRC carcinogenesis as a dynamic, two-stage process, in which the distinction between direct contributors (drivers) and incidental colonizers (passengers) rests on the capacity to initiate primary pathological changes and the timing of entry into the carcinogenic process. Drivers are defined by their capacity to trigger primary alterations at the onset of carcinogenesis (Wang et al., 2024). Passengers are viewed, in the classical model, as opportunistic colonizers: they become enriched in the well-established TME, promote resilience to it, survive under tumor-specific conditions, stabilize dysbiosis, and sustain chronic inflammation (Hajishengallis et al., 2012; Wang et al., 2020; Avril and DePaolo, 2021). This distinction, however, has proven difficult to maintain in practice. Recent metagenomic and multi-omics studies have refined this model, demonstrating that the driver-passenger dichotomy is not always strict. Carcinogenesis involves complex transitional states and network-like microbial interactions (Dejea et al., 2018; Tomkovich et al., 2019; Glazunova et al., 2025; Hu et al., 2025; Bautista et al., 2026).

In contrast to the classical view that driver bacteria act only at the onset of carcinogenesis, bacteria with tumor-initiating potential may continue to exert carcinogenic effects across later stages by adapting to changing tumor conditions (Bullman et al., 2017; Lopès et al., 2020; Piccinno et al., 2025; Bautista et al., 2026). They may contribute to tumor promotion and progression at later stages (Yachida et al., 2019; Li et al., 2021; Seely et al., 2022; Bautista et al., 2026) and, in some cases, combine these abilities with metastatic potential (Yachida et al., 2019; Saito et al., 2025). This persistence across stages complicates the binary classification of microbial roles in CRC (Ascandari et al., 2026). We propose designating taxa that retain tumor-promoting activity beyond the initiation step as secondary drivers, distinct from primary drivers (or “alpha-drivers”) whose activity is largely confined to early carcinogenic events.

Beyond this, a number of clinically relevant bacteria traditionally classified as “passengers” may not only appear at the expected stage of CRC progression (Kumar et al., 2017; Wirbel et al., 2019; Incognito et al., 2025; Piccinno et al., 2025) but also serve as early CRC markers (Kumar et al., 2017; Avril and DePaolo, 2021; Li et al., 2021; Tozzi et al., 2025). Importantly, the classical model defines “passenger” status not only by the degree of pathogenicity but also by the timing of colonization (they are expected to arrive after drivers) (Tjalsma et al., 2012).

Some taxa play both driver-like and passenger-like roles depending on the evolving community structure, the metabolite profiles of the TME, and tumor genetic and immune context (Avril and DePaolo, 2021; Glazunova et al., 2025; Incognito et al., 2025; Bautista et al., 2026). Certain traditional “passenger” taxa may exhibit driver functional virulence profiles and act as late-stage accelerators and active contributors under specific conditions, such as tumor hypoxia or an acidic microenvironment (Boleij et al., 2015; Drewes et al., 2017; Li et al., 2021; Tozzi et al., 2025; Bautista et al., 2026). Such taxa are sometimes termed active passengers (Alexander et al., 2018; Scott et al., 2019), early passengers (Wang et al., 2020), or pro-inflammatory passengers (Avril and DePaolo, 2021), and the assumed indirectness of their role is often far from clear-cut (Paduraru et al., 2025).

Importantly, metagenomic data show that distinct microbial shifts emerge at early and intermediate stages such as carcinoma *in situ* and adenomas (Yachida et al., 2019; Minot et al., 2024; Piccinno et al., 2025), changing throughout tumor development (Duvallet et al., 2017; Liang et al., 2017; Xie et al., 2017; Yachida et al., 2019; Gao et al., 2021). Communities progressively replace one another through multiple transitional phases without fixed states, making driver-passenger boundaries highly fluid (Duvallet et al., 2017; Scott et al., 2019; Avril and DePaolo, 2021; Li et al., 2021; Higashi et al., 2023; Gu et al., 2024; Glazunova et al., 2025). Within a polymicrobial carcinogenic consortium, drivers and passengers collaborate synergistically (Geng et al., 2014). This sustained coexistence further obscures the functional distinction between tumorigenic and opportunistic taxa. The boundary between passive colonization and causal contribution (direct or indirect advantage to the tumor itself) is defined by the existence of a reproducible pro-carcinogenic effect when the taxon is present. Passengers are classically presumed to exert functional influence only at advanced stages of progression (Tjalsma et al., 2012), yet potentially capable of indirectly contributing to carcinogenesis to a greater or lesser extent (Alexander et al., 2018; Scott et al., 2019; Wang et al., 2020; Avril and DePaolo, 2021; Paduraru et al., 2025). The monolithic term “passenger” requires a more precise definition regarding the degree and indirectness of its functional impact (Alexander et al., 2018; Paduraru et al., 2025). Direct versus indirect involvement in CRC is distinguished by the primary target of the taxon: the epithelium and mucosal barrier itself, or secondary environmental components. Nevertheless, the indirectness of certain passenger taxa involvement and the extent of their promoting effects on altered cells, the TME, and driver taxa remain a matter of debate and require further investigation.

Drivers at any stage of progression are fundamentally characterized by their independent capacity to directly affect the host: epithelial and protective mucus layer damage, malignant transformation of healthy epithelial cells (Dejea et al., 2018; Raskov et al., 2018; Haghi et al., 2019; Avril and DePaolo, 2021; Khodaverdi et al., 2021; Périchon et al., 2022). Passenger taxa lack this capacity, yet some more than others become critical mediators, indirectly triggering stepwise shifts in carcinogenic community composition and thereby accelerating tumor progression (Nakatsu et al., 2015; Garza et al., 2020; Périchon et al., 2022). It remains unclear whether carcinogenesis would be arrested in their absence. Given the mutational burden already accumulated by tumor cells, complete arrest is unlikely. However, these taxa may be critically required for efficient and rapid tumor progression, occupying an intermediate position between drivers and classical passengers yet representing a biologically significant link (Kumar et al., 2017; Cheng et al., 2020; Li et al., 2021). We therefore propose that such taxa and the associated functional profiles mediating indirect activity be designated as “enhancers” of driver organisms or of tumorigenesis.While host interindividual variation in microbiota composition makes it difficult to define universal driver-passenger boundaries, the functional mechanisms underlying these roles are more conserved. Virulence factor activity may therefore provide a more reliable criterion for driver-passenger classification than taxon identity alone (Drewes et al., 2017; Mathlouthi et al., 2022). Analyzing disease-taxa-virulence factors relationships becomes essential to determine which specific virulence determinants genuinely matter for particular stages and phenotypes of colorectal carcinogenesis (Wirbel et al., 2019; Mathlouthi et al., 2022; Piccinno et al., 2025). Virulome-level analyses further suggest that distinct virulence factors and virulence signatures are preferentially associated with specific phases of the adenoma-carcinoma sequence, supporting a stage-dependent remodeling of microbial functional potential (Mathlouthi et al., 2022).

This positions the virulome as the operational unit of driver-passenger classification, shifting the analytical focus from community composition to functional categories.

**Figure 1:**
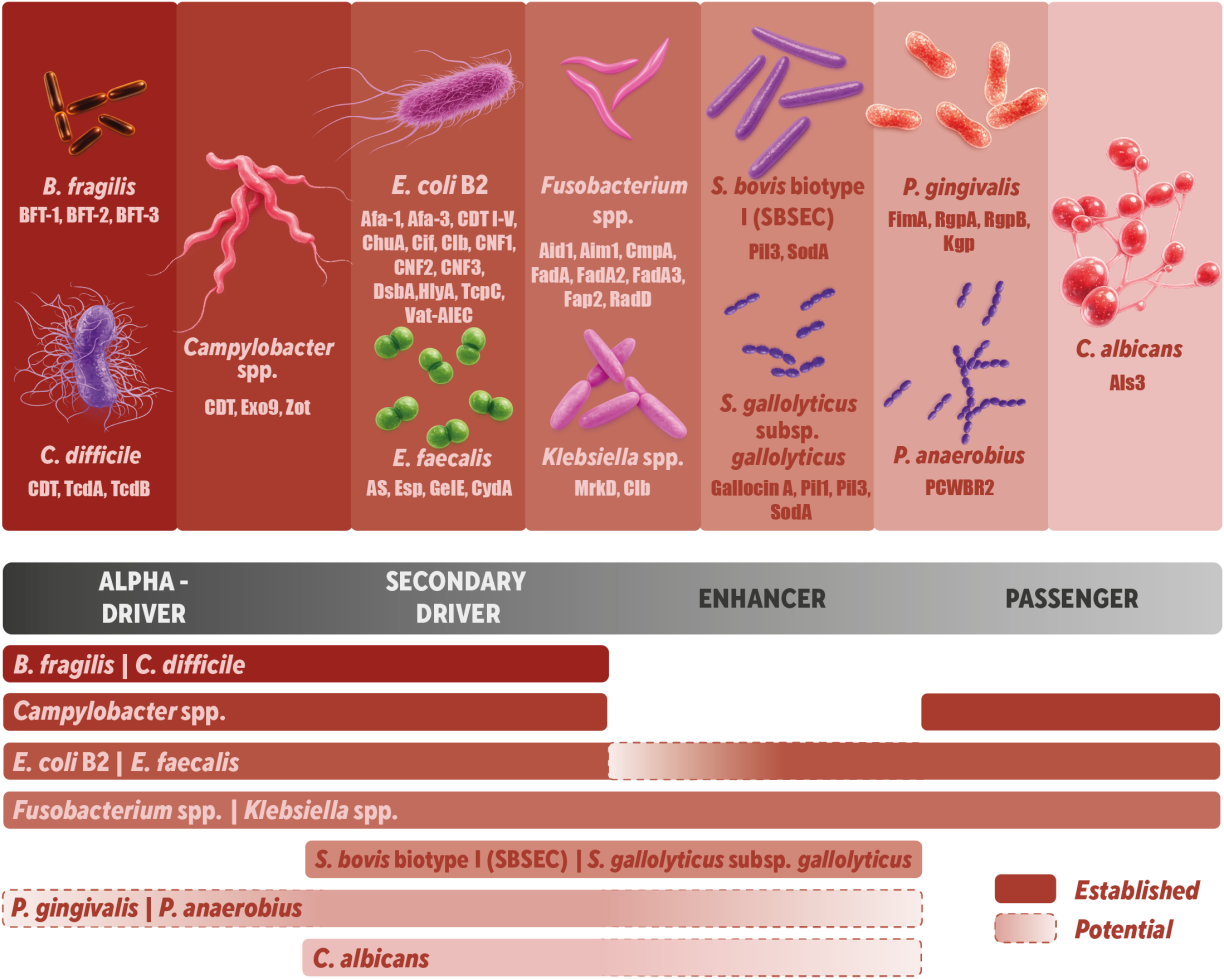
Classification of CRC-associated microorganisms within the expanded driver-passenger framework. The upper panel lists representative taxa and selected associated virulence determinants. The lower panel summarizes their proposed positions along the alpha-driver – secondary driver – enhancer – passenger continuum based on the functional effects of these determinants. Solid bars denote roles supported by current evidence, whereas dashed or semi-transparent bars denote potential roles requiring further validation. Assignments are stage-, strain-, and context-dependent and should be interpreted as functional rather than fixed taxonomic categories.

However, pleiotropy exists at both the organismal and factor levels, with distinct biological effects mediated through different molecular targets and pathways. **Figure 1** illustrates the functional pleiotropy of key CRC-associated microorganisms and their virulence factors across alpha-driver, secondary driver, enhancer, and passenger categories within the proposed expanded driver-passenger framework.

Importantly, taxa referenced within this annotation are characterized exclusively through their associated factors rather than assigned a definitive classification. For example, designating a taxon as carrying enhancer-associated determinants does not exclude its potential role as an active late-stage driver. It reflects only the functional profile identified in the context of CRC-associated factors described in this review.

## CRC-associated virulence determinants in the driver-passenger framework

Critically, function classification is stage-specific: the same virulence determinant may initiate tumorigenesis in early stages, sustain promotion and progression and stabilize niches in late-stage CRC. Capturing this complexity requires extending the driver-passenger framework to the level of individual virulence factors functions, their mechanisms, and their biological effects.

**Figure 2:**
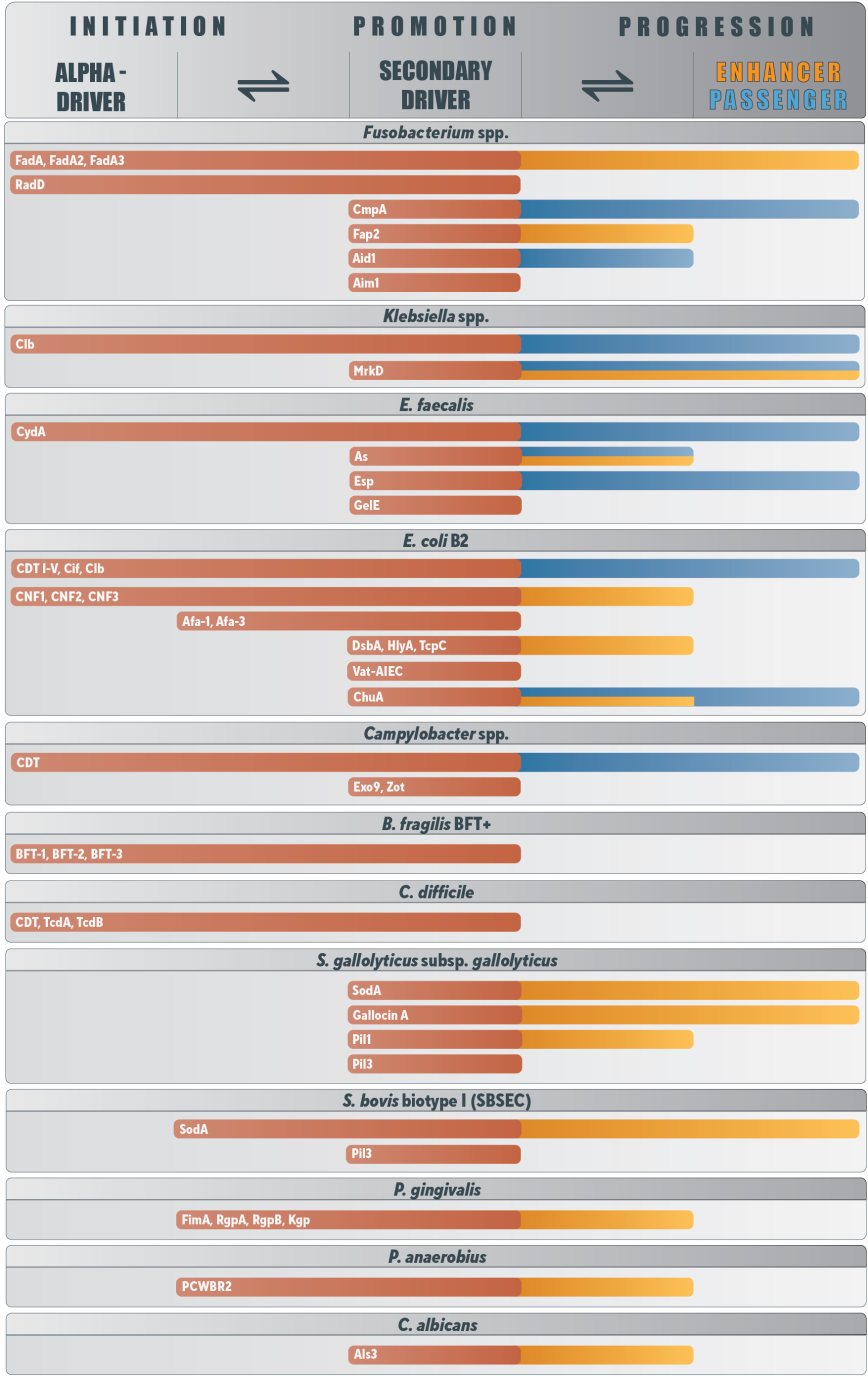
Stage-specific distribution of key CRC-associated virulence determinants within the expanded driver-passenger framework. The figure summarizes selected virulence determinants grouped by representative carrier taxa and maps their proposed functional roles across the initiation, promotion, and progression phases of colorectal carcinogenesis. Horizontal bars indicate the stage range and driver-passenger category in which each determinant may contribute. Red bars denote alpha-driver or secondary driver activity, orange bars denote enhancer activity, and blue bars denote passenger-associated or late-stage persistence-related activity. Multicolored bars indicate pleiotropic determinants with distinct effects across different stages or biological contexts. The assignments reflect current evidence and should be interpreted as stage-, strain-, and context-dependent rather than as fixed properties of either the taxon or the virulence determinant.

The biological role of a virulence factor is determined not only by its intrinsic function but also by host context, disease stage, and polymicrobial interactions. Within the expanded driver-passenger framework, alpha-driver virulence determinants generate the primary carcinogenic hit to the healthy epithelium and shape the TME, stimulating early persistent mucosal alterations, barrier disruption, and formation of a pathogenic community (Hajishengallis et al., 2012; Thiele Orberg et al., 2017; He et al., 2019; Pleguezuelos-Manzano et al., 2020; Kushwaha et al., 2025). Secondary driver virulence determinants do not initiate cancer but act as progression facilitators, delivering tumor-promoting subsequent direct hits. Enhancer factors of carcinogenic progression observed across determinants of both drivers and for those taxa that are conventionally classified, though debated, as canonical passengers act within more or less established TME, conferring resilience and survival under tumor-specific conditions, and indirectly driving tumor progression (Hajishengallis et al., 2012; Nakatsu et al., 2015; Bullman et al., 2017; Drewes et al., 2017; Dejea et al., 2018; Avril and DePaolo, 2021; Wronowska et al., 2025; Bautista et al., 2026). **Figure 2** shows the stage-specific distribution of key CRC-associated virulence factors across functional categories within the continuum of colorectal carcinogenesis.

Virulence factors are rarely unifunctional. Therefore, driver-passenger classification cannot be assigned to factors alone but must be decomposed to the level of individual effects on the host. To apply the proposed factor-function-focused framework, we next summarize key virulence determinants, their core functions, pro-oncogenic effects, and assigned roles within the driver-passenger continuum (see **Supplementary Figure 1**, **Supplementary Table 1**).

This structured annotation refocuses attention on underlying processes, enables estimation of oncogenic potential for individual factors and their synergies in single-organism and polymicrobial settings, and provides a structural basis for causality assessment, risk stratification, biomarker development, and hypothesis modeling.

**Figure 3:**
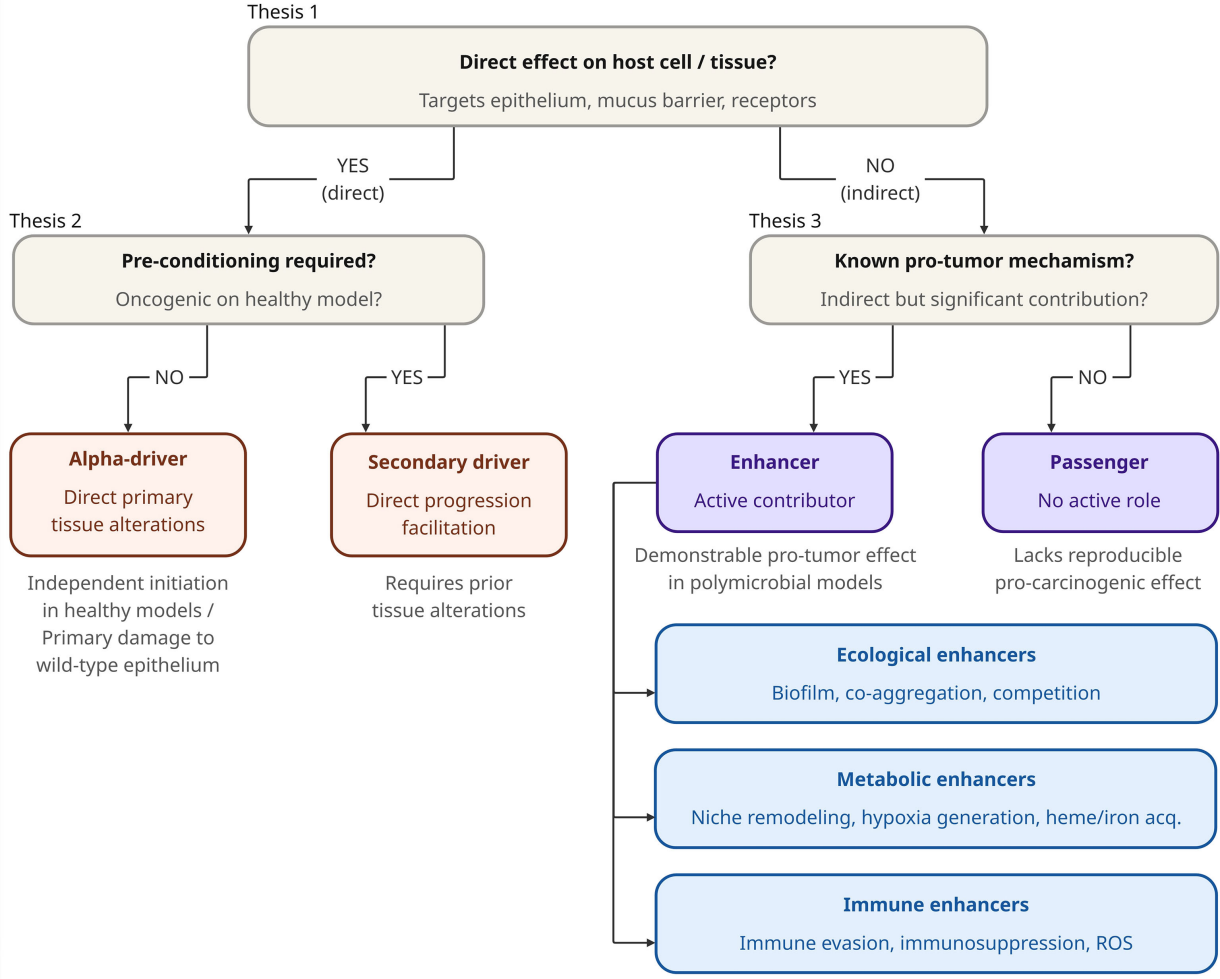
Stepwise decision algorithm for virulence-factor classification within the expanded driver-passenger model. Classification begins by determining whether the factor exerts a direct effect on host cells or tissues. Factors capable of inducing primary oncogenic alterations in an otherwise healthy model are assigned alpha-driver potential, whereas factors requiring pre-existing tissue alterations are classified as secondary drivers. Indirect effects with a reproducible pro-tumor contribution are assigned to the enhancer category and may be further subdivided into ecological, metabolic, and immune mechanisms. Factors without a demonstrated reproducible pro-carcinogenic effect are classified as passenger-associated.

To support the practical applicability of the expanded driver-passenger framework and provide clear operational definitions for the independent classification of novel virulence factors, we propose a stepwise decision algorithm (**Figure 3**). The algorithm comprises three sequential theses, each of which evaluates a specific criterion to determine the functional status of a given determinant, virulence function, or organism. First, this algorithm operationalizes the distinction between direct and indirect host targeting by determining whether a determinant primarily targets the host epithelium itself or secondary environmental components. Second, it differentiates alpha-drivers from secondary drivers based on whether the effect is initiating or progression-dependent. Finally, to account for the diversity of active facilitators of tumor progression, the algorithm divides the ‘enhancer’ category into three mechanistically distinct subgroups: ecological, metabolic, and immune-modulating.

### Alpha-driver determinants

The primary driver profiles are defined by genotoxins causing DNA damage, cyclomodulins inducing cell cycle arrest and polyploidy, determinants provoking oxidative stress, evasion of apoptosis, epigenetic alterations, and disruption of intercellular junctions (Wirbel et al., 2019; Pleguezuelos-Manzano et al., 2020; Avril and DePaolo, 2021; Casimiro-Soriguer et al., 2022; Mathlouthi et al., 2022; Gaab et al., 2023). These factors, through their collective action, subsequently drive cellular senescence with a secretory phenotype (SASP), genomic instability, and hyperproliferation, leading to mutation accumulation and a tumor-promoting microenvironment. At the initiation stage, genotoxins and cytotoxins become key effectors. Alpha-driver factors include: colibactin (Clb) of *Enterobacteriaceae*; cycle inhibiting factor (Cif), five types of cytolethal distending toxin (CDT-I, CDT-II, CDT-III, CDT-IV, CDT-V), cytotoxic necrotizing factors (CNF1, CNF2, CNF3) of different *E. coli* pathotypes; *B. fragilis* toxins (BFT-1, BFT-2, BFT-3); *Clostridioides difficile* toxins (CDT, Toxin A, Toxin B); the cytolethal distending toxin (CDT) of *Campylobacter* spp.; *Fusobacterium* spp. adhesins A (FadA, FadA2, FadA3, RadD); cytochrome bd ubiquinol oxidase subunit I (CydA) of *E. faecalis* (for the references see **Supplementary Table 1**).

### Secondary driver determinants

The principal effect of secondary driver determinants is the induction of downstream changes that further drive tumor promotion and progression. Secondary driver potential includes inflammation-mediated genotoxicity, maintenance and amplification of genomic instability, barrier disruption reinforcement, immune evasion and suppression, proliferation stimulation, angiogenesis, epigenetic reinforcement of malignant phenotypes, metabolic reprogramming, chemoresistance development, and promotion of tissue invasion and metastasis, and expansion and dominance of mature polymicrobial biofilms. The gut microbiota actively drives colorectal carcinogenesis by perturbing critical host cellular mechanisms, most notably autophagy and the epithelial-mesenchymal transition (EMT) (Vescovo et al., 2025). For instance, outer membrane adhesin proteins of the *F. nucleatum* group shield tumor cells from immune attack (Gur et al., 2015), promote disruption of epithelial barrier, invasion (Rubinstein et al., 2013; Abed et al., 2016), host cell motility (Casasanta et al., 2020), and host epithelial adhesion (Fardini et al., 2011). Surface protein PCWBR2 (*P. anaerobius*) markedly enhances proliferation. *E. faecalis g*elatinase GelE (a zinc-dependent enzyme that breaks down large protein structures by hydrolysis of the peptide bonds) degrades extracellular matrix and stimulates invasive and migratory phenotypes in colon cancer cells. Due to their pleiotropic nature, certain classical primary driver virulence factors can operate as secondary drivers; among the best-characterized examples, BFT toxin triggers cell proliferation, oncogenic gene expression, and tumor growth (for the references see **Supplementary Table 1**).

### Non-canonical driver determinants with pathogenic potential

Beyond canonical, readily gene-attributable virulence factors, pathogenic potential across colorectal carcinogenesis is also shaped by toxic metabolites, metabolic adaptations, surface-associated components, and ecological strategies that damage the host epithelium, remodel the tumor microenvironment, or promote persistence within the microbial community (Scott et al., 2019; Avril and DePaolo, 2021).

Reactive oxygen species (ROS), hydrogen sulfide, and superoxides induce epithelial DNA damage, chromosomal instability, and inflammation. These effects represent driver mechanisms. *E. faecalis* produces extracellular superoxide (O_2_^-^) and hydrogen peroxide (H_2_O_2_) via respiration or specific enzyme systems (Huycke et al., 2002; Wang and Huycke, 2007). *P. anaerobius* also induces ROS formation (Tsoi et al., 2017). pks^+^ E. coli causes oxidative stress at an early stage of CRC (Veziant et al., 2016). ETBF induces epithelial spermine oxidase expression, generating ROS and DNA damage (Goodwin et al., 2011). Sulfate-reducing *Desulfovibrio* spp. and certain *Fusobacterium* spp. strains produce genotoxic and pro-inflammatory H_2_S (Wolf et al., 2022; Lin et al., 2023). High concentrations of secondary bile acids modulate the tumor microenvironment and bacterial virulence.

Surface proteins and LPS can trigger oncogenic signaling cascades (Chen et al., 2017; Mu et al., 2020; Casimiro-Soriguer et al., 2022). Strategic metabolic switching occurs: *F. nucleatum* sensu stricto in the presence of *S. sanguinis* ceases fructose consumption - an act of altruistic metabolism that drives streptococcal proliferation and maintains stable biofilm symbiosis (Bibek and Wu, 2024). Mucolytic activity can also be considered a virulence-associated process. Mucin-metabolizing bacteria degrade the protective mucus layer, thereby facilitating epithelial access and providing nutrients, including for CRC-associated pathogens, and have been associated with distinct stages of carcinogenesis [*A. muciniphila* (Png et al., 2010), *B. thetaiotaomicron* (Glazunova et al., 2025), *B. bifidum*, *Ruminococcus gnavus* (Png et al., 2010), *R. torques* (Png et al., 2010; Glazunova et al., 2025)].

### Enhancers of carcinogenic progression

Throughout carcinogenesis, and particularly at the stages of CRC promotion and progression, tumorigenesis is mediated not only by direct but also by significant indirect effects, which prove critical for successful tumor development (Bautista et al., 2026). We classify these mechanisms and active entities as carcinogenic enhancers. Due to their multi-level participation in tumorigenesis, such enhancers can be specific factors, or even their distinct functional effects. Their targets may encompass either driver taxa or other components of the tumor microenvironment.

The roles of enhancers include mediation of oxidative stress; immune protection via cytochrome bd-oxidase (*E. faecalis*), superoxide dismutase (*Streptococcus* spp.), and oxidoreductase (*E. coli*); facilitation of bacterial colonization via pilus adherence factors 1 and 3 (*Streptococcus* spp.); tumor microenvironment adaptation via heme acquisition factor ChuA (*E. coli* pathotype AIEC) and through Fap2, FadA, FadA2, FadA3 (*Fusobacterium* spp.). Early consortium members with passenger-like adaptive traits can persist even at late stages. *F. animalis* C2 (Wirbel et al., 2019) shows acid tolerance and metabolizes nutrients specific to the inflamed intestinal environment (Zepeda-Rivera et al., 2024). *S. gallolyticus* produces bacteriocin Gallocin A. Its activity is enhanced by secondary bile acids enriched in the TME, enabling elimination of closely related streptococci, including SBSEC members (Harrington et al., 2021; Proutière et al., 2023), and enterococci, including *E. faecalis*. Microorganisms with genotoxic activity or DNA-damaging potential, including colibactin-producing and CNF1-positive *E. coli*, *Campylobacter* spp. (especially *C. jejuni* and *C. concisus*), *E. faecalis*, and *C. albicans*, remain active. Synergistic interactions of *F. nucleatum* with ETBF and pks^+^ *E. coli* likely enable these species to persist within the gradually changing carcinogenic community (Dejea et al., 2018; Clay et al., 2022; Paduraru et al., 2025; Bautista et al., 2026). This could explain the frequent detection of *F. nucleatum* (Bullman et al., 2017; Li et al., 2021; Shigematsu et al., 2024a), *B. fragilis* (Bullman et al., 2017; Chang et al., 2025; Saito et al., 2025), and occasionally pks^+^ *E. coli* in metastases (Bertocchi et al., 2021; Marongiu et al., 2021; Gaab et al., 2023; Gu et al., 2024; Shigematsu et al., 2024b; Liu et al., 2025). Beyond these, metastatic sites harbor *F. necrophorum* (Bullman et al., 2017), *C. jejuni* (He et al., 2024), and more predictably, typical early and late conventional “passengers” *B. fragilis*, *B. thetaiotaomicron* (Bullman et al., 2017), *Streptococcus* spp (Gu et al., 2024)., *P. micra*, *Akkermansia muciniphila*, and *P. intermedia* (Bullman et al., 2017; Gu et al., 2024).

### Biofilm-forming determinants

Biofilm-associated factors (adhesins, invasins, pili) (Casimiro-Soriguer et al., 2022) accumulate alongside determinants mediating the aforementioned secondary driver functions (Avril and DePaolo, 2021; Mathlouthi et al., 2022; Bautista et al., 2026) as well as proteolysis, iron/heme acquisition (Mathlouthi et al., 2022) and competitor suppression (Dalmasso et al., 2014).

Virulence-mediated carcinogenic synergy between organisms operates not only at the taxonomic level, but extends to the coordinated action of individual virulence determinants. This synergy of virulence factors within polymicrobial interactions (Zhang et al., 2013; Drewes et al., 2017; Dejea et al., 2018; Bautista et al., 2026) prompts a shift from a single-actor model to the concept of a “consortium of driver determinants”. Within biofilms, interspecies interactions amplify the effects of individual virulence factors beyond simple additive effects, enabling a single species both to support other pathogenic taxa and to directly promote carcinogenesis. Dynamic community interactions occur through coaggregation factors, interspecies activation and regulation, environmental modification, immune and spatial protection, and strategic altruism (cooperative metabolic adjustment, “public goods” production). ETBF exemplifies functional cooperation between CRC-associated taxa: BFT disrupts the mucus layer (Glazunova et al., 2025) and intercellular contacts (Cheng et al., 2020), thereby facilitating epithelial exposure to a second carcinogenic agent, such as colibactin-producing (Dejea et al., 2018; Clay et al., 2022) or adherent-invasive *E. coli* (Glazunova et al., 2025). The combined effect amplifies tumorigenesis through inflammation, activation of proliferation (Cheng et al., 2020), induction of specific mutational signatures [e.g., in the *APC* gene of epithelial cells (Pleguezuelos-Manzano et al., 2020)]. *C. albicans* multifunctional adhesin Als3 mediates tight attachment of *P. gingivalis*, stimulating the production of *P. gingivalis* gingipains that in turn facilitates fungal epithelial invasion (Wronowska et al., 2025). This yeast also enhances *E. faecalis* barrier disruption and cytotoxicity (Maharshak et al., 2015; Kapitan et al., 2025). Beyond these interactions, *C. albicans* actively consumes oxygen, generating hypoxic (anaerobic) conditions (Wronowska et al., 2025), depletes carbohydrate availability, weakening intestinal cells, and thereby facilitating sustained inflammation and a permissive niche for colonization by other organisms (Wang et al., 2025; Zhou et al., 2025). In polymicrobial biofilms, *Treponema denticola*, *P. micra*, and *Prevotella* spp. (e.g., *P. intermedia*) act as enhancers (Meuric et al., 2013; Barbosa et al., 2015; Higashi et al., 2023; Löwenmark et al., 2024), potentiating partner pathogenicity. *T. denticola* (Meuric et al., 2013) and *P. micra* promote *P. gingivalis* biofilm formation and upregulate its virulence genes *kgp* and *rgpA* (Neilands et al., 2019), whereas *P. micra* (Horiuchi et al., 2020) and *Prevotella* spp (Okuda et al., 2012). stabilize coaggregates, particularly with *F. nucleatum* group.

## Methodological considerations and future directions for clinical virulome profiling

Although taxonomic markers show diagnostic utility (Wirbel et al., 2019; Gaab et al., 2023), species and individual strains composition varies between patients and populations. Also, the taxon-based analysis often incompletely captures mechanistic aspects of carcinogenesis (Casimiro-Soriguer et al., 2022; Wu et al., 2023; Minot et al., 2024). Taken together, this may lead to limited reliability. In contrast, in certain contexts, such as early carcinogenesis, functional and metabolic potential profile analysis (KEGG, MetaCyc, eggNOG) outperforms taxonomic-based approaches, accurately distinguishing healthy samples from adenomas and differentiating small adenomas from larger ones (Scott et al., 2019; Clos-Garcia et al., 2020; Mathlouthi et al., 2022; Piccinno et al., 2025). As part of metabolic profiling, virulome analysis also carries a fundamental advantage – direct biological interpretability.

Virulence gene signatures tend to be more stable and reproducible diagnostic markers, independent of bacterial carrier identity (Burns et al., 2015; Thomas et al., 2019; Wirbel et al., 2019). Indeed, the widespread horizontal gene transfer (HGT) demonstrates why taxonomy alone often fails to capture the true oncogenic potential of a microbiome. For instance, the *pks* pathogenicity island encoding the genotoxin colibactin has been found to spread across multiple phylogenetically distant *Enterobacteriaceae* (Pope et al., 2019; Clay et al., 2022). Under conditions of intestinal inflammation, *E. coli* actively promotes the transfer of pathogenicity islands and plasmids carrying *cnf2* gene (transmissible plasmids pVir or pCNF2) (Pérès et al., 1997; Van Bost et al., 2003; Johnson et al., 2010) and *hlyCABD* operon (conjugative plasmid pEO5) (Burgos et al., 2009; Burgos and Beutin, 2010), while genes for CIF, CDT I and CDT IV factors are localized on lambdoid or P2 prophages within the chromosome and are acquired via phage transduction (Samba-Louaka et al., 2009; Onlen Guneri et al., 2022). The genes encoding afimbrial adhesins, such as Afa-1 and Afa-3, demonstrate high mobility. Specifically, the *afa-3* gene cluster is flanked by IS1 insertion sequences, enabling its translocation from plasmid to chromosome as an intact unit through site-specific recombination (Garcia et al., 1994; Onlen Guneri et al., 2022). The binary toxin CDT of *C. difficile* is encoded within a distinct chromosomal locus (CdtLoc) which exhibits features characteristic of a mobile genetic element (Bouvet and Popoff, 2008; Monot et al., 2015; Ramírez-Vargas et al., 2018; Pourliotopoulou et al., 2024). In *E. faecalis*, the transfer of aggregation substance (AS) relies on pheromone-responsive conjugative plasmids pCF10, pAD1 and pAM373 which are exchanged under conditions of close bacterial contact (An and Clewell, 2002; Hirt et al., 2005; Breuer et al., 2018; Vickerman and Mansfield, 2019). Within polymicrobial biofilms, HGT is further facilitated by physical proximity. Across *Fusobacterium* species (*F. polymorphum*, *F. nucleatum*, *F. animalis*, *F. vincentii*), the transfer and extensive mosaic recombination of genes of virulence factors FadA, Fap2, RadD, and CmpA occur naturally within multi-species biofilms, facilitated by the close physical proximity of bacterial cells (Crowley et al., 2024; Hong et al., 2024). Biofilm conditions also enable the exchange of FimA I and FimA IV alleles among *P. gingivalis* strains through natural genetic competence (uptake of free environmental DNA) (Kerr et al., 2014). On a chromosomal level, enterotoxigenic *B. fragilis* mobilizes the *bft* gene via a conjugative transposon (Franco, 2004; Pierce and Bernstein, 2016).

Furthermore, the widespread horizontal gene transfer (HGT) of the pathogenic elements provides substantive rationale for virulome-level classification, making species identity alone a poor proxy for pathogenic potential.

Pathogenicity-associated functions are therefore central to two clinically relevant questions: how a microorganism contributes to carcinogenic progression and at which stage this contribution occurs. Accordingly, virulence determinants themselves become candidate diagnostic and prognostic targets, indicating early oncogenic potential, established tumors, or persistent progressive microbial communities (Mathlouthi et al., 2022; Wang et al., 2022; Shi et al., 2025).

For instance, rather than asking only “Is *F. nucleatum* present?”, it is necessary to consider what this taxonomic designation actually encompasses. Recent revisions of the *F. nucleatum* complex have reassigned several historical subspecies to distinct species, while the name “*F. nucleatum*” may still be used inconsistently to denote either the broader complex (sensu lato) or *F. nucleatum* sensu stricto (Zepeda-Rivera et al., 2025). Species-resolved approaches, including full-length 16S rRNA nanopore sequencing, have enabled discrimination among CRC-associated *Fusobacterium* lineages (Rosenbaum et al., 2026). However, comparative mapping of virulence traits across the redefined complex indicates that some canonical virulence determinants are shared by multiple species, whereas others show lineage-specific distribution (Wolf et al., 2026). Thus, increasingly precise taxonomic resolution identifies the microbial carrier but does not by itself define its carcinogenic functional potential.

The question therefore becomes: “Which virulence-associated genes of *F. nucleatum* does the metagenome carry, individually or in combination?” What matters is the consortium’s functional and virulence potential (genotoxicity, cyclomodulation, biofilm formation, immune suppression, invasion, etc.), which taxa execute it, and by what mechanisms.

To answer this question in a clinically meaningful way, the functional shift from taxonomic detection to virulence profiling must be matched by appropriate analytical tools. From a clinical implementation perspective, shotgun metagenomics, although currently the principal approach for identifying virulence-associated genes, should be viewed primarily as a discovery tool rather than as a final routine diagnostic platform. In low-biomass fecal samples from early-stage CRC or precancerous lesions, and particularly in tissue samples with substantial host DNA contamination, shotgun sequencing may have limited sensitivity for low-abundance virulence determinants and limited precision for quantitative assessment without extensive normalization. A feasible translational workflow may therefore involve initial NGS-based identification of CRC-associated virulence-factor candidates, followed by targeted quantification of selected markers by qPCR or digital PCR in large, standardized cohorts (Liang et al., 2017; Osman et al., 2021; Saito et al., 2025; Shi et al., 2025). Such assays could provide a more sensitive, accessible, and reproducible approach for evaluating known virulence determinants across adenoma, early CRC, advanced CRC, and metastatic disease (Osman et al., 2021). However, this strategy also depends on the completeness and quality of reference databases. Poorly annotated, strain-specific, non-canonical, or previously unrecognized virulence determinants may be missed, making database expansion and curation an essential prerequisite for robust virulome-based diagnostics (Drewes et al., 2017; Queen et al., 2022; Paduraru et al., 2025).

The evidentiary threshold, however, differs between diagnostic screening and causal interpretation. For purely diagnostic purposes, it may be sufficient to demonstrate that different clinical states are reproducibly distinguished by the presence or abundance of DNA markers corresponding to selected virulence determinants. In contrast, mechanistic conclusions require substantially stronger evidence, because detection of a virulence gene does not necessarily imply its transcription, protein production, spatial proximity to host tissue, or biological activity. This distinction is particularly relevant for fecal metagenomics, which primarily reflects luminal and shed microbial DNA and may not fully capture mucosa-adherent or tumor-associated communities where virulence factors exert direct effects on host tissues. Therefore, causal claims regarding the contribution of specific determinants to carcinogenesis should be supported, where possible, by complementary metatranscriptomic, metaproteomic, metabolomic, and spatially resolved approaches (Dejea et al., 2013; Drewes et al., 2017; Lamaudière et al., 2023). This is particularly important because virulence determinants detected in bulk fecal or tissue samples may originate from organisms located in distinct ecological compartments, including the tumor surface, mucus layer, crypts, invasive front, necrotic regions, or adjacent mucosa (Raskov et al., 2018; Zwinsová et al., 2021; He et al., 2024; Bautista et al., 2026).

To address these gaps, several open questions critical for translating accumulated knowledge into practice must be answered:

1. Which microbial functions are critical (necessary and sufficient) to carcinogenic initiation?
2. What causal links exist between specific virulence determinant combinations, in single organisms and across communities, and CRC risk?
3. Is it feasible to link community functional profiles to distinct carcinogenic stages and carcinogenesis specificity? This is complicated by the fact that a bacterium’s virulence factor profile and its cumulative potential strongly influence, but do not fully determine, persistence across tumor stages.
4. What drives competitive displacement within the tumor microenvironment? How do passenger taxa outcompete drivers, given that driver-associated virulence factors themselves confer passenger-like potential? Yet not all drivers are displaced at the late stage, some persist through to metastases, and the mechanisms underlying this selective retention remain unclear.
5. This raises a further question: do passengers harbor determinants with anti-driver activity? Intratumoral microbe-microbe interactions and their influence on each other’s potential remain understudied relative to microbe-host dynamics.
6. Do passengers carry late-stage functional determinants with diagnostic potential?
7. Do drivers functionally transition within mature TME or relocate to peripheral niches (Geng et al., 2014; Garza et al., 2020; Paduraru et al., 2025)?

The association between specific virulence factors and CRC remains under investigation. Most studies document virulence signature enrichment in the tumor metagenome, yet few systematically assess their diagnostic value (Wirbel et al., 2019; Mathlouthi et al., 2022; Gaab et al., 2023). Large-scale, standardized, multicenter studies that examine virulence determinants as independent biomarker candidates across carcinogenesis are notably lacking. Under these conditions, conclusions regarding clinical applicability of such markers remain speculative. Notably, the potency of functionally similar virulence factors, or of comparable functions across different factors and carriers, can vary considerably. Direct comparison of virulence determinant effects as absolute values is not always feasible. This variation stems from carrier spatial localization (aberrant crypt foci, tumor surface, regions with active invasion), host genotype and physiological state, factors co-regulation, and dependencies on other factors.

Metagenomic profiling of fecal samples of CRC patients across different stages, with a focus on virulence-associated genes and examining their relationships with stage of carcinogenesis, may help clinically validate the driver-like or passenger-like potential of specific factors, linking their occurrence to disease state.

## Conclusion

This review proposes a function-focused refinement of the driver-passenger model in CRC, in which microbial roles are defined not by taxonomic identity alone but by the stage-specific activity of virulence determinants, their host effects, and their interactions within polymicrobial communities. The same microorganism may occupy different positions along the driver-enhancer-passenger continuum depending on strain-level features, virulence-factor repertoire, tumor stage, host context, and the structure of the surrounding microbial consortium. Likewise, individual virulence determinants are rarely unifunctional; their biological relevance depends on the specific effect considered, the carrier organism, spatial localization within the tumor niche, and cooperation with other microbial or host-derived factors. This virulome-centered view therefore provides a more mechanistic framework for interpreting CRC-associated microbiota than taxon-based classification alone.

The proposed model also has direct translational implications. Diagnostically, it supports a shift from isolated taxonomic markers toward integrated functional signatures based on selected virulence determinants, their combinations, and the community contexts in which they occur. NGS-based profiling may be used to discover candidate virulence markers, whereas targeted qPCR or digital PCR panels may provide a practical strategy for validating and quantifying known determinants across adenoma, early CRC, advanced CRC, and metastatic disease. Such approaches could improve risk stratification, stage-specific detection, prognostic assessment, and identification of persistent progressive microbial communities. However, diagnostic use should be distinguished from causal interpretation: the detection of virulence-gene DNA may be sufficient for marker development, but mechanistic conclusions require evidence of expression, protein production, spatial localization, and biological activity. Furthermore, full realization of this model requires addressing fundamental open questions regarding intratumoral competitive displacement, potential anti-driver activity of passengers, and the causal links between specific virulence factor combinations and CRC risk.

Therapeutically, this framework suggests that CRC-associated microbial communities should be considered not only as compositional abnormalities but as structured, functionally cooperative ecosystems. Therefore, rational intervention may require targeted disruption of key virulence functions, adhesion mechanisms, biofilm architecture, metabolic cooperation, immune-evasion pathways, or specific carriers of clinically relevant virulence determinants. Such strategies may include anti-virulence approaches, selective inhibition of microbial adhesion and coaggregation, phage-based or probiotic-based modulation, and other precision microbiome interventions aimed at reducing carcinogenic functional potential without broad, dysbiosis-promoting antimicrobial pressure.

Overall, moving from the question of which taxa are present to which virulence functions are active, where they operate, and how they interact provides a conceptual bridge between microbiome association studies and clinically actionable CRC microbiome research. This factor-function framework may support more precise biomarker development, improve interpretation of stage-specific microbial signatures, and guide future therapeutic strategies targeting the functional architecture of CRC-associated polymicrobial consortia.
