# Supplementary material for: Virulome over taxonomy: refining the driver-passenger model in colorectal carcinogenesis
Source: Front Cell Infect Microbiol. 2026 Jul 14;16:1858868. doi: 10.3389/fcimb.2026.1858868 (PMC13407646; doi:10.3389/fcimb.2026.1858868)
Supplement: Supplementary file 1 [file DataSheet1.pdf]

Supplementary Material

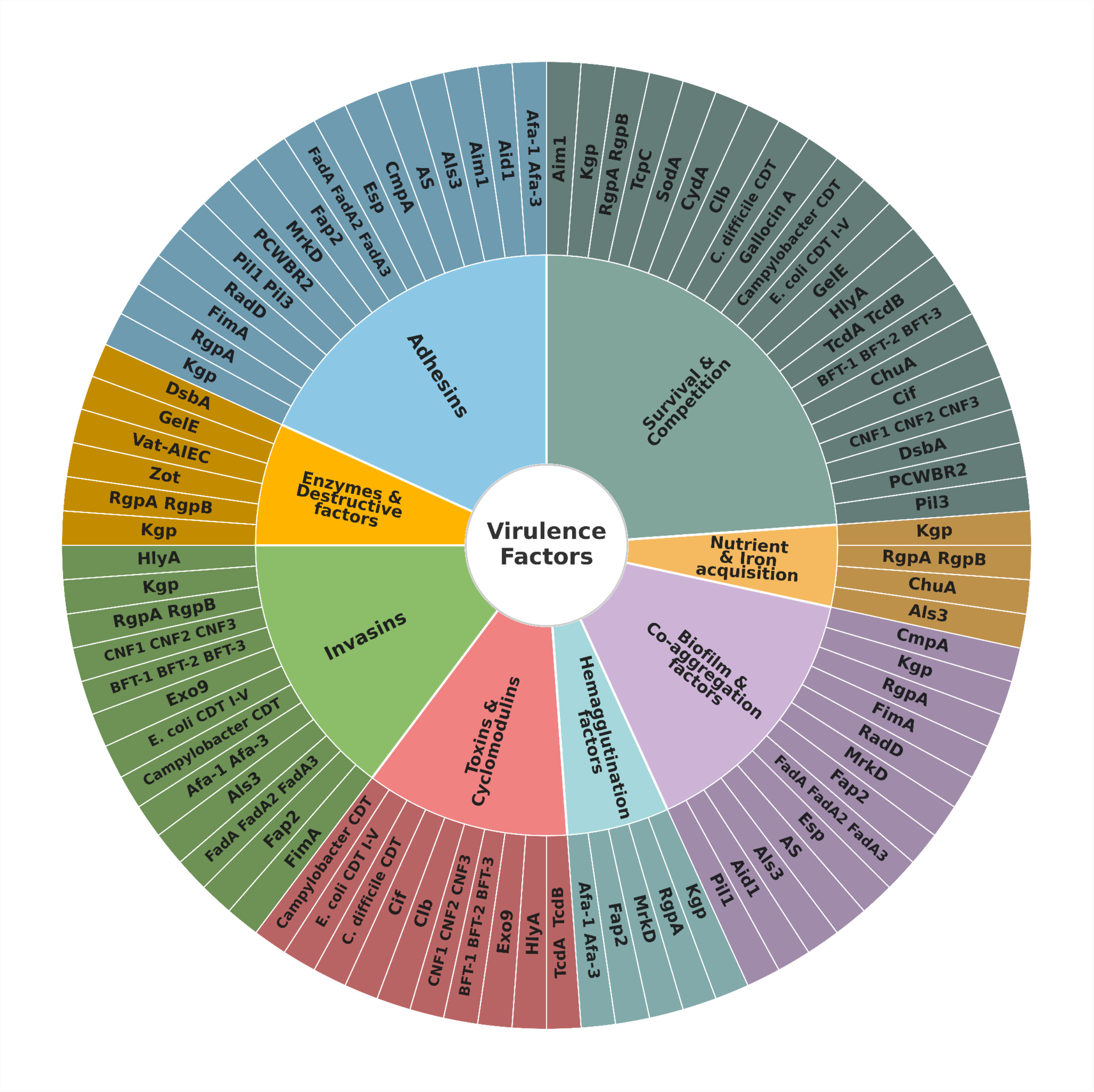

Supplementary Figure 1. Functional classification of CRC-associated virulence factors. The inner ring shows the major functional categories assigned to the virulence factors included in Supplementary Table S1, whereas the outer ring lists the individual determinants assigned to each category. The size of each inner sector reflects the number of factor-function assignments within that category and should not be interpreted as an estimate of prevalence, expression level, or biological effect size. Individual virulence factors may appear in more than one category because of their pleiotropic and context-dependent functions.

Supplementary Table 1. Function-based annotation of CRC-associated virulence determinants within the driver-passenger continuum. The table summarizes key virulence determinants associated with colorectal carcinogenesis, their core functions, pro-oncogenic effects on the host, corresponding driver-passenger roles for individual effects, and consolidated functional classification. Since virulence factors are often multifunctional, assignments were made at the level of specific host effects rather than entire factors.

Virulence Factor/Entities	Functional categories as per Supplementary Fig. 1	Core Functions	Pro-oncogenic mechanisms and effects	Corresponding Driver-Passenger Role	Consolidated Driver-Passenger Role
**Afa-1, Afa-3**<br>*E. coli* (pathotype AIEC, DAEC, EPEC, afaC+, haemagglutinin-positive, afa strains)	Adhesins<br>Invasins<br>Hemagglutination factors	— Mannose-resistant adhesion (1)<br>— Diffuse adhesion via DAF/CD55 (1)<br>— Invasion of GI epithelial cells (2)<br>— Responsible for Dr hemagglutinin (3)	Induction of VEGF expression, promoting angiogenesis and tumor growth	Secondary driver	Secondary driver with alpha-driver potential
			DAF/CD55 (SCR3-domain)-dependent activation of inflammation and neutrophil transepithelial migration	Alpha-driver / Secondary driver	
			Promotion of barrier disruption	Alpha-driver / Secondary driver	
			Promotion of tissue invasion	Secondary driver	
			Modulation of tumor microenvironment via CD55 (DAF) and CEACAM receptors	Secondary driver	
**Aid1**<br>*F. nucleatum* sensu stricto<br>*F. animalis*<br>*F. vincentii*<br>*F. polymorphum*	Adhesins<br>Biofilm and Co-aggregation factors	— RadD-dependent oral interspecies co-aggregation (4)<br>— Biofilm formation and modulation (4)<br>— Interbacterial interactions (4)	Enhancement of chronic inflammation and tumor growth	Secondary driver	Secondary driver with passenger potential
			Promotion of synergistic action of partner genotoxins and other virulence factors	Secondary driver	
			Promotion of microbial translocation	Secondary driver / Passenger	
**Aim1**<br>*F. animalis* clade C1 and clade C2<br>*F. nucleatum* sensu stricto	Adhesins<br>Survival and Competition	— Induction of T-cell apoptosis (5)<br>— Host immune evasion (6)<br>— Modulation host immune response (5)	Impairment of anti-tumor immune surveillance	Secondary driver	Secondary driver
			Promotion of chronic inflammation	Secondary driver	

Virulence Factor/Entities	Functional categories as per Supplementary Fig. 1	Core Functions	Pro-oncogenic mechanisms and effects	Corresponding Driver-Passenger Role	Consolidated Driver-Passenger Role
**Als3**	Adhesins<br>Invasins<br>Biofilm and Co-aggregation factors<br>Nutrient and Iron acquisition	— Adhesion to GI epithelial cells (7)<br>— Invasin-mediated endocytosis (8)<br>— Ferritin binding for iron acquisition (9)<br>— Interbacterial co-aggregation and biofilm formation (10)	Induction of mucosal IgA and Th17-skewed responses	Secondary driver	Secondary driver with enhancer potential
			Promotion of chronic inflammation	Secondary driver	
			Promotion of barrier disruption	Secondary driver	
C. albicans			Promotion of tissue invasion	Secondary driver	
			Promotion of microbial translocation	Secondary driver / Enhancer	
			Remodeling of the tumor microenvironment (hypoxia) and stimulating metastasis via VEGF	Secondary driver	
**AS**	Adhesins<br>Biofilm and Co-aggregation factors	— Adhesion to GI epithelial cells (or urinary tract), enterocytes and neutrophil granulocytes (11)<br>— Interbacterial co-aggregation and biofilm formation through increased hydrophobicity (12)<br>— Host immune evasion (13)<br>— Quorum sensing modulation (14)	Promotion of microbial translocation	Secondary driver / Passenger	Secondary driver with enhancer and passenger potential
			Promotion of barrier disruption	Secondary driver	
Enterococcus spp. plasmid-specific (in particular *E. faecalis*)<br>*E. faecalis* (reactive oxygen species)			Promotion of inflammation via activation of NF-κB and cytokines (IL-6, TNF-α)	Secondary driver	
			Suppression of the "respiratory burst" (release of ROS by macrophages)	Secondary driver / Enhancer	
**Gallocin A**	Survival and Competition	— Gram-positive species growth suppression (15)<br>— Facilitation of bacterial colonization (15)	Remodeling of the tumor microenvironment via promotion of bacteremia	Enhancer	Secondary driver Enhancer
S. gallolyticus subsp. *gallolyticus*			Promotion of IL-8/COX-2-mediated inflammation, epithelial proliferation and tumor growth	Secondary driver	

Virulence Factor/Entities	Functional categories as per Supplementary Fig. 1	Core Functions	Pro-oncogenic mechanisms and effects	Corresponding Driver-Passenger Role	Consolidated Driver-Passenger Role
***Campylobacter* CDT**	Toxins and Cyclomodulins<br>Invasins<br>Survival and Competition	— Host cell invasion (16)<br>— Host immune evasion (17)<br>— Niche formation for colonization (16)	Induction of DNA double-strand breaks via DNase activity and tumor growth	Alpha-driver	Alpha/Secondary driver<br>Passenger
			Promotion of tissue damage: G2/M cell-cycle arrest and apoptosis in epithelial and immune cells	Alpha-driver	
C. coli<br>*C. showae*<br>*C. jejuni*<br>*Campylobacter* spp.			Host immune suppression	Secondary driver	
			Promotion of IL-8-driven chronic inflammation	Secondary driver	
			Promotion of chronic infection	Passenger	
			Activation of JAK2-STAT3-MMP9 leading to metastasis	Secondary driver	
***E. coli* CDT I-V**	Toxins and Cyclomodulins<br>Invasins<br>Survival and Competition	— Host cell invasion (18)<br>— Host immune evasion (19)<br>— Niche formation for colonization (20)	Induction of DNA double-strand breaks via DNase activity and tumor growth	Alpha-driver	Alpha/Secondary driver<br>Passenger
			Promotion of tissue damage: G2/M cell-cycle arrest and apoptosis in epithelial and immune cells	Alpha-driver	
E. coli phylogroup B2			Promotion of genetic instability and tumor growth in precancerous cells (APC/p53 defective)	Alpha-driver	
			Inhibition of macrophage phagocytosis, T/B cell apoptosis, and Ig production.	Secondary driver	
			Host immune suppression	Secondary driver	
			Promotion of chronic inflammation	Secondary driver	
			Promotion of chronic infection	Passenger	
**ChuA**	Nutrient and Iron acquisition<br>Survival and Competition	— Hemin/hemoglobin–iron complexes uptake for iron acquisition (23)<br>— Colonization of inflamed host niches (24)<br>— Evasion of nutritional immunity (23)	Promotion of macrophage invasion, granuloma formation and release of TNF-α, IL-8 and IL-12	Secondary driver / Enhancer	Secondary driver<br>Passenger with enhancer potential
E. coli (pathotype AIEC)			Promotion of dysbiosis	Passenger	

Virulence Factor/Entities	Functional categories as per Supplementary Fig. 1	Core Functions	Pro-oncogenic mechanisms and effects	Corresponding Driver-Passenger Role	Consolidated Driver-Passenger Role
C. difficile **CDT**	Toxins and Cyclomodulins<br>Survival and Competition	— Disruption of host cytoskeleton (21)<br>— Inhibition of actin polymerization (21)<br>— Promotion of adherence via formation of microtubule-rich protrusions (22)	Disruption of epithelial actin filaments leading to cell death	Alpha-driver<br>Secondary driver	Alpha/Secondary driver
			Promotion of barrier disruption	Secondary driver	
			Activation of Wnt/β-catenin signaling and a pro-tumorigenic immune response	Alpha-driver / Secondary driver	
C. difficile (CDT+ pathotype)			Promotion of IL-6/ IL-8/IL-1β/IL-18–driven inflammation	Secondary driver	
			Impairment of cell signaling and survival by suppressing the PI-3K/Akt/pGSK3β pathway	Alpha-driver	
			Promotion of chromosomal instability	Alpha-driver	
			Promotion of cell migration and metastasis via JAK2/STAT3/MMP9 activation	Secondary driver	
**Cif**	Toxins and Cyclomodulin<br>Survival and Competition	— Host cell cycle manipulation (25)<br>— Host immune indirect evasion (26)<br>— Host immune indirect response modulation (27)<br>— Niche formation for colonization (26)	Induction of G1/S and G2/M cell-cycle arrest via p21/p27 accumulation	Alpha-driver	Alpha/Secondary driver<br>Passenger
			Induction of delayed apoptosis	Alpha-driver / Secondary driver	
E. coli phylogroup B2 (in particular pathotype EPEC)			Promotion of cytoskeletal rearrangements via Rho GTPase deregulation	Secondary driver	
			Activation of pro-inflammatory signaling cascades	Secondary driver	
			Promotion of chronic infection	Passenger	
**Clb**	Toxins and Cyclomodulins<br>Survival and Competition	— Host immune evasion (28)<br>— Host immune response modulation (28)<br>— Niche formation for colonization (29)<br>— Secondary interbacterial growth suppression (30)	Induction of DNA damage via interstrand crosslinks, genomic instability, and tumor growth	Alpha-driver	Alpha/Secondary driver<br>Passenger
E. coli phylogroups A, B2, B1, D (pathotypes AIEC and EPEC)<br>*K. pneumonia*<br>*K. aerogenes*<br>*C. koseri*<br>*E. hormaechei*<br>*E. cloacae*			Induction of G2/M cell-cycle arrest	Alpha-driver	
			Induction of inducing vigorous proliferation via senescence and SASP	Alpha-driver / Secondary driver	
			Host immune suppression	Secondary driver	
			Promotion of chronic infection	Passenger	
			Chemoresistance	Secondary driver	

Virulence Factor/Entities	Functional categories as per Supplementary Fig. 1	Core Functions	Pro-oncogenic mechanisms and effects	Corresponding Driver-Passenger Role	Consolidated Driver-Passenger Role
**CmpA**<br>*F. nucleatum* sensu stricto<br>*F. animalis* clade C2	Adhesin<br><br>Biofilm and Co-aggregation factors	— Arginine-inhibitable coaggregation and biofilm formation (31)	Promotion of chronic infection	Passenger	Secondary driver<br>Passenger
			Host immune suppression	Secondary driver	
**CNF1, CNF2, CNF3**<br><br>*E. coli* phylogroup B2	Toxins and Cyclomodulins<br><br>Invasins<br><br>Survival and Competition	— Host cell cycle manipulation (32)<br>— Host immune evasion (33)<br>— Host immune response modulation (34)<br>— Disruption of epithelial barrier (35)<br>— Promotion of host cell motility (36)<br>— Promotion of invasion (37)<br>— Induction of inflammation directly and indirectly and establishment of a stable niche (34)	Activation of Rho GTPases with actin remodeling, increasing epithelial/tumor cell motility and invasiveness (CNF2 prefers RhoA/Rac1)	Secondary driver	Alpha/Secondary driver with enhancer potential
			Promotion of G2/M cell-cycle arrest with macrocytosis and failed cytokinesis, leading to aneuploidy and chromosomal instability	Alpha-driver	
			Promotion of release of proinflammatory IL-1β, IL-6, IL-8 and immunosuppressive IL-10	Secondary driver	
			Promotion of immunomodulation	Secondary driver	
			Promotion of NF-κB/COX-2-mediated pro-inflammatory, anti-apoptotic signaling and tumor growth	Secondary driver	
			Promotion of barrier disruption	Alpha-driver / Secondary driver	
			Promotion of tissue invasion	Secondary driver	
			Stimulation of the ROS production	Secondary driver / Enhancer	
			Stimulation of acquisition of phagocytic properties (macropinocytosis)	Secondary driver / Enhancer	
			Promotion of tumor invasiveness and metastatic potential	Secondary driver	
**DsbA**<br>*E. coli* phylogroup B2 (in particular pathotypes AIEC, EPEC)	Enzymes and Destructive factors<br><br>Survival and Competition	— Immune evasion factor (indirectly) (38)<br>— Biogenesis of exported proteins (39)<br>— Type III secretion system assembly factor (40)	Promotion of chronic inflammation	Secondary driver	Secondary driver with enhancer potential
			Promotion of macrophage invasion, granuloma formation and release of TNF-α, IL-8 and IL-12	Secondary driver / Enhancer	
			Suppression and remodeling of macrophage function	Secondary driver / Enhancer	

Virulence Factor/Entities	Functional categories as per Supplementary Fig. 1	Core Functions	Pro-oncogenic mechanisms and effects	Corresponding Driver-Passenger Role	Consolidated Driver-Passenger Role
**Esp**<br>*E. faecalis*	Adhesins<br>Biofilm and Co-aggregation factors	— Adhesion to GI epithelial cells (41)<br>— Biofilm formation and strengthening (42)<br>— Host immune modulation (43)	Promotion of chronic infection	Passenger	Secondary driver<br>Passenger
			Promotion of chronic inflammation	Secondary driver	
			Promotion of barrier disruption	Secondary driver	
			Promotion of immune evasion	Secondary driver	
			Stimulation of angiogenesis via PI3K/AKT/mTOR (VEGFA, IL-8)	Secondary driver	
**BFT-1, BFT-2, BFT-3**<br>ETBF	Toxins and Cyclomodulins<br>Invasins<br>Survival and Competition	— Disruption of epithelial barrier (44)<br>— Promotion of host cell motility (45)<br>— Facilitation of bacterial colonization (46)<br>— Induction of local inflammation and indirect modulation of host immune response (47)<br>— Establishment of a stable niche (48)	Promotion of barrier disruption and necrosis via E-cadherin cleavage and tight-junction loss	Secondary driver	Alpha/Secondary driver
			Promotion of Wnt/β-catenin-driven proliferative signaling, oncogenic gene expression and tumor growth via proteolytic degradation of E-cadherin complexes	Alpha-driver	
			Activation of the p38 MAPK/AP-1 pathway (driving cell proliferation and IL-8 secretion)	Secondary driver	
			Activation of the STAT3/Th17/NF-κB immune-inflammatory axis	Secondary driver	
			Host immune suppression	Secondary driver	
			Inhibition of apoptosis of damaged cells	Secondary driver	
			Promotion of neutrophil-rich inflammation	Secondary driver	
			Generation of oncogenic E-cadherin fragments and ROS	Alpha-driver / Secondary driver	
			Promotion of tissue invasion	Secondary driver	
**Exo9**<br>*C. concisus* (AICC)	Toxins and Cyclomodulins<br>Invasins	— Host cell cycle manipulation (49)<br>— Promotion of invasion (50)	Promotion of actin cytoskeleton remodeling	Secondary driver	Secondary driver
			Promotion of chronic inflammation	Secondary driver	
			Promotion of barrier disruption	Secondary driver	
			Induction of epithelial cell cytotoxicity or stress	Secondary driver	

Virulence Factor/Entities	Functional categories as per Supplementary Fig. 1	Core Functions	Pro-oncogenic mechanisms and effects	Corresponding Driver-Passenger Role	Consolidated Driver-Passenger Role
**FadA**<br>*F. nucleatum* sensu stricto<br>*F. hwasookii*<br>*F. varium*<br>*F. necrophorum*	Adhesins<br>Invasins<br>Biofilm and Co-aggregation factors	— Host epithelial and endothelial adhesion (51)<br>— Disruption of epithelial barrier (51)<br>— Promotion of host cell motility and invasion (52)<br>— Induction of inflammation directly and indirectly (53)<br>— Modulation of host tumor microenvironment (54)<br>— Establishment of a stable niche (55)<br>— Interbacterial co-aggregation and biofilm formation (55)	Promotion of Wnt/β-catenin-driven colorectal cells proliferation, oncogenic gene expression and tumor growth via direct E-cadherin binding	Alpha-driver / Secondary driver	Alpha/Secondary driver<br>Enhancer
			Blocking macrophage apoptosis by activating the ERK pathway (in conjunction with PI3K)	Secondary driver / Enhancer	
			Promotion of NF-κB–mediated chronic mucosal inflammation	Secondary driver	
			Induction of DNA damage	Alpha-driver	
			Remodeling of the tumor microenvironment	Enhancer	
			Promotion of barrier disruption via E- and VE-cadherin	Secondary driver	
			Promotion of tissue invasion	Secondary driver	
**FadA2**<br>*F. varium*<br>*F. ulcerans*<br>*F. animalis* clade C1 and clade C2	Adhesins<br>Invasins<br>Biofilm and Co-aggregation factors	Similar to FadA functions, but with reduced effect (55), (56)	Promotion of Wnt/β-catenin-driven colorectal cells proliferation, oncogenic gene expression and tumor growth via direct E-cadherin binding	Alpha-driver / Secondary driver	Alpha/Secondary driver<br>Enhancer
			Blocking macrophage apoptosis by activating the ERK pathway (in conjunction with PI3K)	Secondary driver / Enhancer	
			Promotion of NF-κB–mediated chronic mucosal inflammation	Secondary driver	
			Induction of DNA damage	Alpha-driver	
			Remodeling of the tumor microenvironment	Enhancer	
			Promotion of barrier disruption via E- and VE-cadherin	Secondary driver	
			Promotion of tissue invasion	Secondary driver	
**GelE**<br>*E. faecalis* (GelE-positive)	Enzymes and Destructive factors<br>Survival and Competition	— Hydrolyzation of gelatin, elastin, collagen, hemoglobin, polymerized fibrin, C3, C3a, C3b, C5a, pheromone-bound proteins (59)<br>— Immune evasion (60)	Promotion of barrier disruption	Secondary driver	Secondary driver
			Promotion of tumor cell motility and migration	Secondary driver	
			Promotion of tissue invasion	Secondary driver	
			Facilitation of metastatic spread	Secondary driver	
			Modulation of immune responses via inactivation of complement components and chemotactic factors	Secondary driver	

Virulence Factor/Entities	Functional categories as per Supplementary Fig. 1	Core Functions	Pro-oncogenic mechanisms and effects	Corresponding Driver-Passenger Role	Consolidated Driver-Passenger Role
**FadA3**<br>*F. varium*<br>*F. ulcerans*	Adhesins<br>Invasins<br>Biofilm and Co-aggregation factors	Similar to FadA functions, but with reduced effect (55), (56)	Promotion of Wnt/β-catenin-driven colorectal cells proliferation, oncogenic gene expression and tumor growth via direct E-cadherin binding	Alpha-driver / Secondary driver	Alpha/Secondary driver Enhancer
			Promotion of NF-κB–mediated chronic mucosal inflammation	Secondary driver	
			Induction of DNA damage	Alpha-driver	
			Remodeling of the tumor microenvironment	Enhancer	
			Promotion of barrier disruption via E- and VE-cadherin	Secondary driver	
			Promotion of tissue invasion	Secondary driver	
**Fap2**<br>*F. nucleatum* sensu stricto<br>*F. animalis* clade C2	Adhesins<br>Invasins<br>Biofilm and Co-aggregation factors<br>Hemagglutination factors	— Adhesion to Gal-GalNAc–expressing epithelial cells (57)<br>— Interbacterial co-aggregation and biofilm formation (58)<br>— Modulation of host tumor microenvironment (6)<br>— Hemagglutination and TIGIT-dependent immune evasion (58)<br>— Facilitation of bacterial invasion and dissemination (57)	Promotion of tumor cell motility and migration via induction of IL-8/CXCL1 secretion	Secondary driver	Secondary driver with enhancer potential
			Remodeling of the tumor microenvironment	Secondary driver / Enhancer	
			TIGIT-dependent T-cell and NK-cell inhibition	Secondary driver	
			Induction of a proinflammatory response by myeloid cells stimulation	Secondary driver	
			Induction of lymphocyte apoptosis	Secondary driver	
			Impairment of anti-tumor immune surveillance	Secondary driver	
**HlyA**<br>*E. coli* phylogroup B2 (in particular hemolytic pathotype UPEC)<br>*P. stuartii*	Toxins and Cyclomodulins<br>Invasins<br>Survival and Competition	— Disruption of epithelial barrier (61)<br>— Host immune response modulation (62)<br>— Promotion of tissue invasion (63)<br>— Induction of inflammation directly and indirectly (64)<br>— Modulation of host tumor microenvironment (61)	Promotion of barrier disruption	Secondary driver	Secondary driver with enhancer potential
			Promotion of tissue invasion	Secondary driver	
			Promotion of increased intestinal permeability	Secondary driver	
			Promotion of chronic inflammation	Secondary driver	
			Remodeling of the tumor microenvironment	Secondary driver / Enhancer	

Virulence Factor/Entities	Functional categories as per Supplementary Fig. 1	Core Functions	Pro-oncogenic mechanisms and effects	Corresponding Driver-Passenger Role	Consolidated Driver-Passenger Role
**CydA**<br><br>*E. faecalis* (reactive oxygen species)	Survival and Competition	— Energy generation via oxygen respiration (65)<br>— Survival and persistence in oxidative niches (65)<br>— Interbacterial growth suppression (66)	Induction of oxidative stress in host cells	Alpha-driver / Secondary driver	Alpha/Secondary driver<br>Passenger
			DNA damage, including double-strand breaks and base modifications	Alpha-driver	
			Potential promotion of DNA-protein cross-linking	Alpha-driver	
			Modulation of tumor microenvironment	Passenger	
			Promotion of chronic inflammation	Secondary driver	
**MrkD**<br><br>*K. oxytoca*<br>*K. michiganensis,*<br>*K. grimontii*<br>*K. pasteurii*<br>*K. spallanzanii*<br>*K. huaxiensis*<br>*K. quasivariicola*<br>*K. pneumonia*<br>*C. freundii,*<br>*C.koseri,*<br>*P. stuartii*	Adhesins<br><br>Biofilm and Co-aggregation factors<br><br>Hemagglutination factors	— Adhesion to GI epithelial cells or ECM (collagen IV/V) (67)<br>— Interbacterial interspecific biofilm formation (68)<br>— Hemagglutination and host immune response modulation (69)	Promotion of chronic inflammation	Secondary driver	Secondary driver<br>Enhancer<br>Passenger
			Remodeling of the tumor microenvironment via ECM engagement	Enhancer	
			Modulation of tumor microenvironment	Passenger	
**PCWBR2**<br><br>*P. anaerobius*	Adhesins<br><br>Survival and Competition	— Host epithelial adhesion and stable colonization facilitation (70)<br>— Promotion of host cell motility and proliferation (70)<br>— Induction of local inflammation (70)<br>— Modulation of host tumor microenvironment (70)	Promotion of PI3K–Akt–FAK–driven proliferation and tumor growth	Alpha-driver / Secondary driver	Secondary driver with alpha-driver and enhancer potential
			Promotion of NF-κB–mediated pro-tumor mucosal inflammation	Secondary driver	
			Promotion of epithelial motility	Secondary driver	
			Remodeling of the tumor microenvironment	Secondary driver / Enhancer	
			Promotion of tissue invasion	Secondary driver	
**Pil1**<br><br>*S. gallolyticus* subsp. *gallolyticus*	Adhesins<br><br>Biofilm and Co-aggregation factors	— Adhesion to colonic collagen (types I and IV) (71)<br>— Promotion of colonization and biofilm formation on collagen-rich host tissues (72)	Promotion of chronic inflammation	Secondary driver	Secondary driver with enhancer potential
			Promotion of macrophage invasion, granuloma formation and release of TNF-α, IL-8 and IL-12	Secondary driver / Enhancer	
			Promotion of barrier disruption	Secondary driver	
			Promotion of tissue invasion	Secondary driver	

Virulence Factor/Entities	Functional categories as per Supplementary Fig. 1	Core Functions	Pro-oncogenic mechanisms and effects	Corresponding Driver-Passenger Role	Consolidated Driver-Passenger Role
**Pil3**	Adhesins<br><br>Survival and Competition	— Adhesion to colonic fibrinogen, mucus (MUC2, MUC5AC) and mucins (73)<br>— Facilitation of bacterial colonization (74)<br>— Host signaling modulation (75)	Promotion of NF-κB → IL-8/IL-1β inflammation	Secondary driver	Secondary driver
S. infantarius subsp. *infantarius,*<br>*S. lutetiensis*<br>*S. equinus.*<br>*S. alactolyticus*<br>*S. gallolyticus* subsp. *gallolyticus*<br>*S. macedonicus*<br>*S. pasteurianus*			Promotion of barrier disruption	Secondary driver	
**RadD**	Adhesins<br><br>Biofilm and Co-aggregation factors	— Host epithelial adhesion and stable colonization facilitation (76)<br>— Polymicrobial co-aggregation and biofilm formation (77)<br>— Induction of T-lymphocyte apoptosis (78)<br>— Disruption of epithelial barrier (79)	Induction of Rho/Rac/Cdc42 inactivation via GT-dependent O-glucosylation	Alpha-driver	Alpha/Secondary driver
			Induction of actin cytoskeleton collapse with epithelial cell rounding and death	Secondary driver	
F. animalis clade C1 and clade C2<br>*F. nucleatum* sensu stricto<br>*F. polymorphum*			Promotion of tight-junction disassembly and barrier disruption	Secondary driver	
			Promotion of NF-κB-dependent inflammation	Secondary driver	
			Suppression of phagocyte functions via Rho GTPase inactivation	Secondary driver	
			Promotion of tissue invasion	Secondary driver	
**Zot**	Enzymes and Destructive factors	— Disruption of epithelial barrier (tight-junction disassembly) (90)<br>— Induction of inflammation (91)<br>— Facilitation of bacterial colonization (92)	Promotion of zonula occludens-dependent barrier disruption	Secondary driver	Secondary driver
C. concisus (AToCC)			Promotion of increased intestinal permeability	Secondary driver	
			Promotion of IL-8-dependent inflammation	Secondary driver	

Virulence Factor/Entities	Functional categories as per Supplementary Fig. 1	Core Functions	Pro-oncogenic mechanisms and effects	Corresponding Driver-Passenger Role	Consolidated Driver-Passenger Role
**SodA**<br>*S. infantarius* subsp. *infantarius,*<br>*S. lutetiensis*<br>*S. equinus*<br>*S. alactolyticus*<br>*S. gallolyticus* subsp. *gallolyticus*<br>*S. macedonicus*<br>*S. pasteurianus*	Survival and Competition	— Protection from reactive oxygen in ROS-rich tumor microenvironment (80)<br>— Facilitation of bacterial colonization (81)<br>— Establishment of a stable niche (81)<br>— Modulation of host tumor microenvironment (80)<br>— Host immune evasion (80)	Remodeling of the tumor microenvironment	Enhancer	Secondary driver<br>Enhancer
			Promotion of inflammation	Secondary driver	
			Generation of oncogenic ROS	Secondary driver	
			NETosis and pyroptosis suppression	Secondary driver	
			Host immune suppression and therapy resistance via suppression of NET-formation in neutrophils	Secondary driver	
**TcdA**<br>*C. difficile* (A+B+ pathotype and A−B+ pathotype)	Toxins and Cyclomodulins<br>Survival and Competition	— Disruption of epithelial barrier (82)<br>— Modulation of host immune responses (82)<br>— Suppression of phagocyte functions via Rho GTPase inactivation (weaker than TcdB) (83)<br>— Induction of local inflammation (84)<br>— Establishment of a stable niche for competitive advantage (82)	Induction of Thr37 RhoA, Thr37 Rac1/Cdc42, Thr35 Rap1 inactivation via UDP-glucose-dependent monoglycosylation	Alpha-driver	Alpha/Secondary driver
			Promotion of Wnt/β-catenin-driven colorectal cells proliferation	Alpha-driver / Secondary driver	
			Induction of actin cytoskeleton collapse with epithelial cell rounding and death	Secondary driver	
			Promotion of tight-junction disassembly and barrier disruption	Secondary driver	
			Promotion of NF-κB-dependent and IL-17-dependent inflammation	Secondary driver	
			Dysregulation of phagocyte functions via Rho GTPase inactivation	Secondary driver	
			Promotion of tissue invasion	Secondary driver	
**Vat-AIEC**<br>*E. coli* (pathotype AIEC)	Enzymes and Destructive factors	— Mucins degradation (89)<br>— Facilitation of bacterial adhesion and colonization (89)	Promotion of barrier disruption	Secondary driver	Secondary driver

Virulence Factor/Entities	Functional categories as per Supplementary Fig. 1	Core Functions	Pro-oncogenic mechanisms and effects	Corresponding Driver-Passenger Role	Consolidated Driver-Passenger Role
**TcdB**	Toxins and Cyclomodulins<br><br>Survival and Competition	— Disruption of epithelial barrier (more potent; broader tropism via defined receptors) (82)<br>— Modulation of host immune responses (strong inflammasome activation) (82)<br>— Suppression of phagocyte functions via Rho GTPase inactivation (stronger than TcdA) (85)<br>— Induction of local inflammation (86)<br>— Establishment of a stable niche for competitive advantage (87)	Induction of Rho/Rac/Cdc42 inactivation via GT-dependent O-glucosylation	Alpha-driver	Alpha/Secondary driver
			Crypt stem cell damage (Lgr5+)	Alpha-driver / Secondary driver	
			Promotion of Wnt/β-catenin-driven colorectal cells proliferation	Alpha-driver / Secondary driver	
			Induction of actin cytoskeleton collapse with epithelial cell rounding and death	Secondary driver	
C. difficile (A+B+ pathotype)			Promotion of tight-junction disassembly and barrier disruption	Secondary driver	
			Induction of inflammasome-driven IL-1β/IL-18 release and inflammation	Secondary driver	
			Promotion of NF-κB/STAT3, IL-17-dependent inflammation	Secondary driver	
			Suppression of phagocyte functions via Rho GTPase inactivation	Secondary driver	
			Promotion of tissue invasion	Secondary driver	
**FimA**	Adhesins<br><br>Invasins<br><br>Biofilm and Co-aggregation factors	— Host epithelial adhesion, invasion and stable colonization facilitation via Gal–GalNAc and extracellular-matrix binding (93)<br>— Interbacterial co-aggregation and biofilm formation (94)<br>— Promotion of host cell proliferation (95)<br>— Host immune evasion (96)	Promotion of proliferation and tumor growth	Secondary driver	Secondary driver with alpha-driver and enhancer potential
			Activation of PI3K/Akt and β-catenin-driven metastasis	Alpha-driver / Secondary driver	
			Promotion of IL-1, IL-6, IL-8 and TNF-α-dependent inflammation	Secondary driver	
P. gingivalis			Promotion of macrophage invasion	Secondary driver / Enhancer	
			Remodeling of mucosal immune surveillance	Secondary driver	
			Promotion of tissue invasion	Secondary driver	

Virulence Factor/Entities	Functional categories as per Supplementary Fig. 1	Core Functions	Pro-oncogenic mechanisms and effects	Corresponding Driver-Passenger Role	Consolidated Driver-Passenger Role
**TcpC**	Survival and Competition	— Host immune evasion (88)<br>— Inhibition of host immune response (88)<br>— Establishment of a stable niche (88)	Suppression of TLR-MyD88-dependent innate immune signaling	Secondary driver	Secondary driver with enhancer potential
E. coli phylogroup B2 (in particular pathotype UPEC)			Immunosuppression via Inhibition of NF-κB activation with reduced pro-inflammatory cytokine production (IL-6, IL-12, TNFα)	Secondary driver	
			Promotion of immune evasion via blockade of TLR-driven inflammation	Secondary driver / Enhancer	
			Suppression of immune response from Th17 lymphocytes	Secondary driver	
**RgpA**	Adhesins<br>Biofilm and Co-aggregation factors<br>Enzymes and Destructive factors<br>Hemagglutination factors<br>Nutrient & Iron acquisition<br>Survival and Competition<br>Invasins	— Disruption of epithelial barrier (97)<br>— Host immune evasion (98)<br>— Host immune response modulation (99)<br>— Induction of inflammation directly and indirectly (99)<br>— Promotion of invasion (97)<br>— Facilitation of bacterial adhesion/coaggregation (100)<br>— Nutrient acquisition (101)<br>— Processing/maturation of bacterial surface proteins (102)	Promotion of barrier disruption	Secondary driver	Secondary driver with alpha-driver and enhancer potential
			Promotion of cell proliferation via NLRP3 inflammasomes and MAPK/ERK activation (including KRAS, BRAF, MEK, ERK, and transcription factor AP1)	Alpha-driver / Secondary driver	
			Induction of apoptosis	Secondary driver	
			Promotion of PAR2/NF-κB-driven inflammation	Secondary driver	
P. gingivalis			Promotion of immune evasion via host immune-response modulation	Secondary driver	
			Promotion of tissue invasion and and metastasis via cleavage of ProMMP-9 to active MMP-9	Secondary driver	
			Degradation of antimicrobial peptides, immunoglobulins, complement, ICAM-1 and cytokines (IL-1β, IL-4, IL-5, IL-6, IL-8, TNF-α)	Secondary driver / Enhancer	
			Stimulation of angiogenesis via IL-6/IL-8-triggered JAK/STAT signaling (leading to VEGF secretion) and upregulation of EFNB2	Secondary driver	

<table>
<tr><th>Virulence Factor/Entities</th><th>Functional categories as per Supplementary Fig. 1</th><th>Core Functions</th><th>Pro-oncogenic mechanisms and effects</th><th>Corresponding Driver-Passenger Role</th><th>Consolidated Driver-Passenger Role</th></tr>
<tr><td rowspan="4">RgpB</td><td rowspan="8">Enzymes and Destructive factors

Survival and Competition

Invasins

Nutrient and Iron acquisition</td><td rowspan="8">— Disruption of mucus barrier (MUC2) (103)
— Disruption of epithelial barrier (104)
— Host immune evasion (105)
— Host immune response modulation (105)
— Induction of inflammation directly and indirectly (106)
— Promotion of invasion (107)
— Nutrient acquisition (108)
— Processing/maturation of bacterial surface proteins (109)</td><td>Promotion of barrier disruption via MUC2 degradation</td><td>Secondary driver</td><td rowspan="8">Secondary driver with alpha-driver and enhancer potential</td></tr>
<tr><td>Promotion of cell proliferation via NLRP3 inflammasomes and MAPK/ERK activation (including KRAS, BRAF, MEK, ERK, and transcription factor AP1)</td><td>Alpha-driver / Secondary driver</td></tr>
<tr><td>Promotion of PAR2/NF-κB-driven and IL-8-dependent inflammation</td><td>Secondary driver</td></tr>
<tr><td>Promotion of immune evasion via host immune-response modulation</td><td>Secondary driver</td></tr>
<tr><td rowspan="4">P. gingivalis</td><td>Promotion of tissue invasion and and metastasis via cleavage of ProMMP-9 to active MMP-9</td><td>Secondary driver</td></tr>
<tr><td>Generation of oncogenic ROS via p38/ERK/PKC</td><td>Alpha-driver / Secondary driver</td></tr>
<tr><td>Degradation of antimicrobial peptides, immunoglobulins, complement, ICAM-1 and cytokines (IL-1β, IL-4, IL-5, IL-6, IL-8, TNF-α)</td><td>Secondary driver / Enhancer</td></tr>
<tr><td>Stimulation of angiogenesis via IL-6/IL-8-triggered JAK/STAT signaling (leading to VEGF secretion) and upregulation of EFNB2</td><td>Secondary driver</td></tr>
</table>

Virulence Factor/Entities	Functional categories as per Supplementary Fig. 1	Core Functions	Pro-oncogenic mechanisms and effects	Corresponding Driver-Passenger Role	Consolidated Driver-Passenger Role
**Kgp**<br><br>*P. gingivalis*	Adhesins<br><br>Biofilm and Co-aggregation factors<br><br>Enzymes and Destructive factors<br><br>Hemagglutination factors<br><br>Nutrient and Iron acquisition<br><br>Survival and Competition<br><br>Invasins	— Disruption of epithelial barrier (97)<br>— Host immune evasion (110)<br>— Host immune response modulation (111)<br>— Induction of inflammation directly and indirectly (111)<br>— Promotion of invasion (112)<br>— Facilitation of bacterial adhesion/coaggregation (113)<br>— Nutrient acquisition (114)	Promotion of barrier disruption	Secondary driver	Secondary driver with alpha-driver and enhancer potential
			Promotion of cell proliferation via NLRP3 inflammasomes and MAPK/ERK activation (including KRAS, BRAF, MEK, ERK, and transcription factor AP1)	Alpha-driver / Secondary driver	
			Promotion of PAR2/NF-κB-driven inflammation	Secondary driver	
			Promotion of immune evasion via host immune-response modulation	Secondary driver	
			Promotion of tissue invasion and and metastasis via cleavage of ProMMP-9 to active MMP-9	Secondary driver	
			Promotion of permeability of blood vessels	Secondary driver	
			Degradation of antimicrobial peptides, immunoglobulins, complement, ICAM-1 and cytokines (IL-1β, IL-4, IL-5, IL-6, IL-8, TNF-α), less effective than RgpA, RgpB	Secondary driver / Enhancer	
			Stimulation of angiogenesis via IL-6/IL-8-triggered JAK/STAT signaling (leading to VEGF secretion) and upregulation of EFNB2	Secondary driver	

References

1. Labigne-Roussel AF, Lark D, Schoolnik G, Falkow S. Cloning and expression of an afimbrial adhesin (AFA-I) responsible for P blood group-independent, mannose-resistant hemagglutination from a pyelonephritic Escherichia coli strain. Infect Immun. 1984 Oct;46(1):251–9. doi:10.1128/iai.46.1.251-259.1984

2. Jouve M, Garcia MI, Courcoux P, Labigne A, Gounon P, Le Bouguénec C. Adhesion to and invasion of HeLa cells by pathogenic Escherichia coli carrying the afa-3 gene cluster are mediated by the AfaE and AfaD proteins, respectively. Infect Immun. 1997 Oct;65(10):4082–9. doi:10.1128/iai.65.10.4082-4089.1997

3. Nowicki B, Labigne A, Moseley S, Hull R, Hull S, Moulds J. The Dr hemagglutinin, afimbrial adhesins AFA-I and AFA-III, and F1845 fimbriae of uropathogenic and diarrhea-associated Escherichia coli belong to a family of hemagglutinins with Dr receptor recognition. Infect Immun. 1990 Jan;58(1):279–81. doi:10.1128/iai.58.1.279-281.1990

4. Kaplan A, Kaplan CW, He X, McHardy I, Shi W, Lux R. Characterization of aid1, a Novel Gene Involved in Fusobacterium nucleatum Interspecies Interactions. Microb Ecol. 2014 Aug;68(2):379–87. doi:10.1007/s00248-014-0400-y

5. Kaplan CW, Lux R, Huynh T, Jewett A, Shi W, Haake SK. *Fusobacterium nucleatum* Apoptosis-inducing Outer Membrane Protein. J Dent Res. 2005 Aug;84(8):700–4. doi:10.1177/154405910508400803

6. Gur C, Ibrahim Y, Isaacson B, Yamin R, Abed J, Gamliel M, et al. Binding of the Fap2 Protein of Fusobacterium nucleatum to Human Inhibitory Receptor TIGIT Protects Tumors from Immune Cell Attack. Immunity. 2015 Feb;42(2):344–55. doi:10.1016/j.immuni.2015.01.010

7. Liu Y, Filler SG. Candida albicans Als3, a Multifunctional Adhesin and Invasin. Eukaryot Cell. 2011 Feb;10(2):168–73. doi:10.1128/EC.00279-10

8. Phan QT, Myers CL, Fu Y, Sheppard DC, Yeaman MR, Welch WH, et al. Als3 Is a Candida albicans Invasin That Binds to Cadherins and Induces Endocytosis by Host Cells. Heitman J, editor. PLoS Biol. 2007 Feb 20;5(3):e64. doi:10.1371/journal.pbio.0050064

9. Almeida RS, Brunke S, Albrecht A, Thewes S, Laue M, Edwards JE, et al. The Hyphal-Associated Adhesin and Invasin Als3 of Candida albicans Mediates Iron Acquisition from Host Ferritin. Mitchell AP, editor. PLoS Pathog. 2008 Nov 21;4(11):e1000217. doi:10.1371/journal.ppat.1000217

10. Klotz SA, Gaur NK, De Armond R, Sheppard D, Khardori N, Edwards JE, et al. *Candida albicans* Als proteins mediate aggregation with bacteria and yeasts. Med Mycol. 2007 Jan;45(4):363–70. doi:10.1080/13693780701299333

11. Lee SA, Liu F, Yuwono C, Phan M, Chong S, Biazik J, et al. Emerging *Aeromonas* enteric infections: their association with inflammatory bowel disease and novel pathogenic mechanisms. Manning SD, editor. Microbiol Spectr. 2023 Oct 17;11(5):e01088-23. doi:10.1128/spectrum.01088-23

12. Chenia HY, Duma S. Characterization of virulence, cell surface characteristics and biofilm‐forming ability of *Aeromonas* spp. isolates from fish and sea water. Journal of Fish Diseases. 2017 Mar;40(3):339–50. doi:10.1111/jfd.12516

13. Garduño RA, Moore AR, Olivier G, Lizama AL, Garduño E, Kay WW. Host cell invasion and intracellular residence by *Aeromonas salmonicida* : Role of the S-layer. Can J Microbiol. 2000 Jul 1;46(7):660–8. doi:10.1139/w00-034

14. Rasch M, Kastbjerg V, Bruhn J, Dalsgaard I, Givskov M, Gram L. Quorum sensing signals are produced by Aeromonas salmonicida and quorum sensing inhibitors can reduce production of a potential virulence factor. Dis Aquat Org. 2007 Dec 13;78:105–13. doi:10.3354/dao01865

15. Proutière A, Du Merle L, Garcia-Lopez M, Léger C, Voegele A, Chenal A, et al. Gallocin A, an Atypical Two-Peptide Bacteriocin with Intramolecular Disulfide Bonds Required for Activity. LaRock CN, editor. Microbiol Spectr. 2023 Apr 13;11(2):e05085-22. doi:10.1128/spectrum.05085-22

16. Biswas D, Fernando U, Reiman C, Willson P, Potter A, Allan B. Effect of Cytolethal Distending Toxin of Campylobacter jejuni on Adhesion and Internalization in Cultured Cells and in Colonization of the Chicken Gut. Avian Diseases. 2006 Dec;50(4):586–93. doi:10.1637/7514-020706R1.1

17. Pratt JS, Sachen KL, Wood HD, Eaton KA, Young VB. Modulation of Host Immune Responses by the Cytolethal Distending Toxin of *Helicobacter hepaticus*. Infect Immun. 2006 Aug;74(8):4496–504. doi:10.1128/IAI.00503-06

18. Tóth I, Nougayrède JP, Dobrindt U, Ledger TN, Boury M, Morabito S, et al. Cytolethal Distending Toxin Type I and Type IV Genes Are Framed with Lambdoid Prophage Genes in Extraintestinal Pathogenic *Escherichia coli*. Infect Immun. 2009 Jan;77(1):492–500. doi:10.1128/IAI.00962-08

19. Bielaszewska M, Sinha B, Kuczius T, Karch H. Cytolethal Distending Toxin from Shiga Toxin-Producing *Escherichia coli* O157 Causes Irreversible G_2 /M Arrest, Inhibition of Proliferation, and Death of Human Endothelial Cells. Infect Immun. 2005 Jan;73(1):552–62. doi:10.1128/IAI.73.1.552-562.2005

20. Jinadasa RN, Bloom SE, Weiss RS, Duhamel GE. Cytolethal distending toxin: a conserved bacterial genotoxin that blocks cell cycle progression, leading to apoptosis of a broad range of mammalian cell lineages. Microbiology. 2011 Jul 1;157(7):1851–75. doi:10.1099/mic.0.049536-0

21. Schwan C, Stecher B, Tzivelekidis T, Van Ham M, Rohde M, Hardt WD, et al. Clostridium difficile Toxin CDT Induces Formation of Microtubule-Based Protrusions and Increases Adherence of Bacteria. Blanke SR, editor. PLoS Pathog. 2009 Oct 16;5(10):e1000626. doi:10.1371/journal.ppat.1000626

22. Schwan C, Kruppke AS, Nölke T, Schumacher L, Koch-Nolte F, Kudryashev M, et al. *Clostridium difficile* toxin CDT hijacks microtubule organization and reroutes vesicle traffic to increase pathogen adherence. Proc Natl Acad Sci USA. 2014 Feb 11;111(6):2313–8. doi:10.1073/pnas.1311589111

23. Torres AG, Payne SM. Haem iron‐transport system in enterohaemorrhagic *Escherichia coli* O157:H7. Molecular Microbiology. 1997 Feb;23(4):825–33. doi:10.1046/j.1365-2958.1997.2641628.x

24. Dogan B, Suzuki H, Herlekar D, Sartor RB, Campbell BJ, Roberts CL, et al. Inflammation-associated Adherent-invasive Escherichia coli Are Enriched in Pathways for Use of Propanediol and Iron and M-cell Translocation: Inflammatory Bowel Diseases. 2014 Nov;20(11):1919–32. doi:10.1097/MIB.0000000000000183

25. Samba-Louaka A, Nougayrède JP, Watrin C, Jubelin G, Oswald E, Taieb F. Bacterial cyclomodulin Cif blocks the host cell cycle by stabilizing the cyclin-dependent kinase inhibitors $p21^{waf1}$ and $p27^{kip1}$. Cellular Microbiology. 2008 Dec;10(12):2496–508. doi:10.1111/j.1462-5822.2008.01224.x

26. Samba-Louaka A, Nougayrède JP, Watrin C, Oswald E, Taieb F. The Enteropathogenic *Escherichia coli* Effector Cif Induces Delayed Apoptosis in Epithelial Cells. Infect Immun. 2009 Dec;77(12):5471–7. doi:10.1128/IAI.00860-09

27. Long TM, Nisa S, Donnenberg MS, Hassel BA. Enteropathogenic Escherichia coli Inhibits Type I Interferon- and RNase L-Mediated Host Defense To Disrupt Intestinal Epithelial Cell Barrier Function. Bäumler AJ, editor. Infect Immun. 2014 Jul;82(7):2802–14. doi:10.1128/IAI.00105-14

28. Lu MC, Chen YT, Chiang MK, Wang YC, Hsiao PY, Huang YJ, et al. Colibactin Contributes to the Hypervirulence of pks+ K1 CC23 Klebsiella pneumoniae in Mouse Meningitis Infections. Front Cell Infect Microbiol. 2017 Mar 31;7. doi:10.3389/fcimb.2017.00103

29. Chen J, Byun H, Liu R, Jung IJ, Pu Q, Zhu CY, et al. A commensal-encoded genotoxin drives restriction of *Vibrio cholerae* colonization and host gut microbiome remodeling. Proc Natl Acad Sci USA. 2022 Mar 15;119(11):e2121180119. doi:10.1073/pnas.2121180119

30. Wong JJ, Ho FK, Choo PY, Chong KKL, Ho CMB, Neelakandan R, et al. Escherichia coli BarA-UvrY regulates the pks island and kills Staphylococci via the genotoxin colibactin during interspecies competition. Peschel A, editor. PLoS Pathog. 2022 Sep 6;18(9):e1010766. doi:10.1371/journal.ppat.1010766

31. Lima BP, Shi W, Lux R. Identification and characterization of a novel *Fusobacterium nucleatum* adhesin involved in physical interaction and biofilm formation with *Streptococcus gordonii*. MicrobiologyOpen. 2017 Jun;6(3):e00444. doi:10.1002/mbo3.444

32. Falzano L, Filippini P, Travaglione S, Miraglia AG, Fabbri A, Fiorentini C. *Escherichia coli* Cytotoxic Necrotizing Factor 1 Blocks Cell Cycle G_2 /M Transition in Uroepithelial Cells. Infect Immun. 2006 Jul;74(7):3765–72. doi:10.1128/IAI.01413-05

33. Miraglia AG, Travaglione S, Meschini S, Falzano L, Matarrese P, Quaranta MG, et al. Cytotoxic Necrotizing Factor 1 Prevents Apoptosis via the Akt/IκB Kinase Pathway: Role of Nuclear Factor-κB and Bcl-2. Isberg R, editor. MBoC. 2007 Jul;18(7):2735–44. doi:10.1091/mbc.e06-10-0910

34. Gall-Mas L, Fabbri A, Namini M, Givskov M, Fiorentini C, Krejsgaard T. The Bacterial Toxin CNF1 Induces Activation and Maturation of Human Monocyte-Derived Dendritic Cells. IJMS. 2018 May 8;19(5):1408. doi:10.3390/ijms19051408

35. Hopkins AM, Walsh SV, Verkade P, Boquet P, Nusrat A. Constitutive activation of Rho proteins by CNF-1 influences tight junction structure and epithelial barrier function. Journal of Cell Science. 2003 Feb 15;116(4):725–42. doi:10.1242/jcs.00300

36. May M, Kolbe T, Wang T, Schmidt G, Genth H. Increased Cell-Matrix Adhesion upon Constitutive Activation of Rho Proteins by Cytotoxic Necrotizing Factors from *E. coli* and *Y. pseudotuberculosis*. Journal of Signal Transduction. 2012 Jul 5;2012:1–10. doi:10.1155/2012/570183

37. Landraud L, Pulcini C, Gounon P, Flatau G, Boquet P, Lemichez E. E. coli CNF1 toxin: a two-in-one system for host-cell invasion. International Journal of Medical Microbiology. 2004;293(7–8):513–8. doi:10.1078/1438-4221-00295

38. Bringer MA, Rolhion N, Glasser AL, Darfeuille-Michaud A. The Oxidoreductase DsbA Plays a Key Role in the Ability of the Crohn's Disease-Associated Adherent-Invasive *Escherichia coli* Strain LF82 To Resist Macrophage Killing. J Bacteriol. 2007 Jul;189(13):4860–71. doi:10.1128/JB.00233-07

39. Coulthurst SJ, Lilley KS, Hedley PE, Liu H, Toth IK, Salmond GPC. DsbA Plays a Critical and Multifaceted Role in the Production of Secreted Virulence Factors by the Phytopathogen Erwinia carotovora subsp. atroseptica. Journal of Biological Chemistry. 2008 Aug;283(35):23739–53. doi:10.1074/jbc.M801829200

40. Ellermeier CD, Slauch JM. RtsA Coordinately Regulates DsbA and the *Salmonella* Pathogenicity Island 1 Type III Secretion System. J Bacteriol. 2004 Jan;186(1):68–79. doi:10.1128/JB.186.1.68-79.2004

41. Lund B, Edlund C. Bloodstream Isolates of *Enterococcus faecium* Enriched with the Enterococcal Surface Protein Gene, *esp* , Show Increased Adhesion to Eukaryotic Cells. J Clin Microbiol. 2003 Nov;41(11):5183–5. doi:10.1128/JCM.41.11.5183-5185.2003

42. Tendolkar PM, Baghdayan AS, Gilmore MS, Shankar N. Enterococcal Surface Protein, Esp, Enhances Biofilm Formation by *Enterococcus faecalis*. Infect Immun. 2004 Oct;72(10):6032–9. doi:10.1128/IAI.72.10.6032-6039.2004

43. Zou J, Shankar N. Surface protein Esp enhances pro-inflammatory cytokine expression through NF-κB activation during enterococcal infection. Innate Immun. 2016 Jan;22(1):31–9. doi:10.1177/1753425915611237

44. Riegler M, Lotz M, Sears C, Pothoulakis C, Castagliuolo I, Wang CC, et al. *Bacteroides fragilis* toxin 2 damages human colonic mucosa in vitro. Gut. 1999 Apr 1;44(4):504–10. doi:10.1136/gut.44.4.504

45. Sears CL. Enterotoxigenic *Bacteroides fragilis* : a Rogue among Symbiotes. Clin Microbiol Rev. 2009 Apr;22(2):349–69. doi:10.1128/CMR.00053-08

46. Hill CA, Casterline BW, Valguarnera E, Hecht AL, Shepherd ES, Sonnenburg JL, et al. Bacteroides fragilis toxin expression enables lamina propria niche acquisition in the developing mouse gut. Nat Microbiol. 2024 Jan 2;9(1):85–94. doi:10.1038/s41564-023-01559-9

47. Kim WS, Hwang S, Gwon SY, Jo M, Yoo SH, Hong J, et al. Bacteroides fragilis Toxin Induces Sequential Proteolysis of E-Cadherin and Inflammatory Response in Mouse Intestinal Epithelial Cell Line. Microorganisms. 2025 Mar 28;13(4):781. doi:10.3390/microorganisms13040781

48. Casterline BW, Hecht AL, Choi VM, Bubeck Wardenburg J. The *Bacteroides fragilis* pathogenicity island links virulence and strain competition. Gut Microbes. 2017 Jul 4;8(4):374–83. doi:10.1080/19490976.2017.1290758

49. Kofol R, Pirs M, Kotar T, Lejko Zupanc T, Celar Šturm A, Kukec A, et al. Genetic Diversity of Campylobacter concisus Isolates from Slovenian Patients with Infectious Diarrhoea. Microorganisms. 2025 Dec 31;14(1):87. doi:10.3390/microorganisms14010087

50. Gemmell MR, Berry S, Mukhopadhya I, Hansen R, Nielsen HL, Bajaj-Elliott M, et al. Comparative genomics of *Campylobacter concisus* : Analysis of clinical strains reveals genome diversity and pathogenic potential. Emerging Microbes & Infections. 2018 Dec;7(1):1–17. doi:10.1038/s41426-018-0118-x

51. Fardini Y, Wang X, Témoin S, Nithianantham S, Lee D, Shoham M, et al. *Fusobacterium nucleatum* adhesin FadA binds vascular endothelial cadherin and alters endothelial integrity. Molecular Microbiology. 2011 Dec;82(6):1468–80. doi:10.1111/j.1365-2958.2011.07905.x

52. Xu M, Yamada M, Li M, Liu H, Chen SG, Han YW. FadA from Fusobacterium nucleatum Utilizes both Secreted and Nonsecreted Forms for Functional Oligomerization for Attachment and Invasion of Host Cells. Journal of Biological Chemistry. 2007 Aug;282(34):25000–9. doi:10.1074/jbc.M611567200

53. Guo P, Tian Z, Kong X, Yang L, Shan X, Dong B, et al. FadA promotes DNA damage and progression of Fusobacterium nucleatum-induced colorectal cancer through up-regulation of chk2. J Exp Clin Cancer Res. 2020 Dec;39(1):202. doi:10.1186/s13046-020-01677-w

54. Rubinstein MR, Wang X, Liu W, Hao Y, Cai G, Han YW. Fusobacterium nucleatum Promotes Colorectal Carcinogenesis by Modulating E-Cadherin/β-Catenin Signaling via its FadA Adhesin. Cell Host & Microbe. 2013 Aug;14(2). doi:10.1016/j.chom.2013.07.012

55. Meng Q, Gao Q, Mehrazarin S, Tangwanichgapong K, Wang Y, Huang Y, et al. *Fusobacterium nucleatum* secretes amyloid‐like FadA to enhance pathogenicity. EMBO Reports. 2021 Jul 5;22(7):e52891. doi:10.15252/embr.202152891

56. Manson McGuire A, Cochrane K, Griggs AD, Haas BJ, Abeel T, Zeng Q, et al. Evolution of Invasion in a Diverse Set of *Fusobacterium* Species. Gilmore MS, editor. mBio. 2014 Dec 31;5(6):e01864-14. doi:10.1128/mBio.01864-14

57. Abed J, Emgård JEM, Zamir G, Faroja M, Almogy G, Grenov A, et al. Fap2 Mediates Fusobacterium nucleatum Colorectal Adenocarcinoma Enrichment by Binding to Tumor-Expressed Gal-GalNAc. Cell Host & Microbe. 2016 Aug;20(2):215–25. doi:10.1016/j.chom.2016.07.006

58. Coppenhagen-Glazer S, Sol A, Abed J, Naor R, Zhang X, Han YW, et al. Fap2 of Fusobacterium nucleatum Is a Galactose-Inhibitable Adhesin Involved in Coaggregation, Cell Adhesion, and Preterm Birth. Camilli A, editor. Infect Immun. 2015 Mar;83(3):1104–13. doi:10.1128/IAI.02838-14

59. Del Papa MF, Hancock LE, Thomas VC, Perego M. Full Activation of *Enterococcus faecalis* Gelatinase by a C-Terminal Proteolytic Cleavage. J Bacteriol. 2007 Dec 15;189(24):8835–43. doi:10.1128/JB.01311-07

60. Park SY, Shin YP, Kim CH, Park HJ, Seong YS, Kim BS, et al. Immune Evasion of *Enterococcus faecalis* by an Extracellular Gelatinase That Cleaves C3 and iC3b. The Journal of Immunology. 2008 Nov 1;181(9):6328–36. doi:10.4049/jimmunol.181.9.6328

61. Schulz E, Schumann M, Schneemann M, Dony V, Fromm A, Nagel O, et al. Escherichia coli Alpha-Hemolysin HlyA Induces Host Cell Polarity Changes, Epithelial Barrier Dysfunction and Cell Detachment in Human Colon Carcinoma Caco-2 Cell Model via PTEN-Dependent Dysregulation of Cell Junctions. Toxins. 2021 Jul 26;13(8):520. doi:10.3390/toxins13080520

62. Wang K, Liao J, Yuan Y, Chen Z, Gou Q, Jing H, et al. Alpha hemolysin enhances the immune response by modulating dendritic cell differentiation via ADAM10-Notch signaling. Sig Transduct Target Ther. 2025 Oct 8;10(1):334. doi:10.1038/s41392-025-02432-3

63. Naskar M, Parekh VP, Abraham MA, Alibasic Z, Kim MJ, Suk G, et al. α-Hemolysin promotes uropathogenic E. coli persistence in bladder epithelial cells via abrogating bacteria-harboring lysosome acidification. Subashchandrabose S, editor. PLoS Pathog. 2023 May 11;19(5):e1011388. doi:10.1371/journal.ppat.1011388

64. Verma V, Kumar P, Gupta S, Yadav S, Dhanda RS, Thorlacius H, et al. α-Hemolysin of uropathogenic E. coli regulates NLRP3 inflammasome activation and mitochondrial dysfunction in THP-1 macrophages. Sci Rep. 2020 Jul 28;10(1):12653. doi:10.1038/s41598-020-69501-1

65. Borisov VB, Gennis RB, Hemp J, Verkhovsky MI. The cytochrome bd respiratory oxygen reductases. Biochimica et Biophysica Acta (BBA) - Bioenergetics. 2011 Nov;1807(11):1398–413. doi:10.1016/j.bbabio.2011.06.016

66. Kana BD, Weinstein EA, Avarbock D, Dawes SS, Rubin H, Mizrahi V. Characterization of the *cydAB* -Encoded Cytochrome *bd* Oxidase from *Mycobacterium smegmatis*. J Bacteriol. 2001 Dec 15;183(24):7076–86. doi:10.1128/JB.183.24.7076-7086.2001

67. Sebghati TA, Clegg S. Construction and Characterization of Mutations within the *Klebsiella* $mrkD_{1P}$ Gene That Affect Binding to Collagen Type V. Moore RN, editor. Infect Immun. 1999 Apr;67(4):1672–6. doi:10.1128/IAI.67.4.1672-1676.1999

68. Childers BM, Van Laar TA, You T, Clegg S, Leung KP. $MrkD_{1P}$ from Klebsiella pneumoniae Strain IA565 Allows for Coexistence with Pseudomonas aeruginosa and Protection from Protease-Mediated Biofilm Detachment. Payne SM, editor. Infect Immun. 2013 Nov;81(11):4112–20. doi:10.1128/IAI.00521-13

69. Sebghati TAS, Korhonen TK, Hornick DB, Clegg S. Characterization of the Type 3 Fimbrial Adhesins of *Klebsiella* Strains. Infect Immun. 1998 Jun;66(6):2887–94. doi:10.1128/IAI.66.6.2887-2894.1998

70. Long X, Wong CC, Tong L, Chu ESH, Ho Szeto C, Go MYY, et al. Peptostreptococcus anaerobius promotes colorectal carcinogenesis and modulates tumour immunity. Nat Microbiol. 2019 Sep 9;4(12):2319–30. doi:10.1038/s41564-019-0541-3

71. Danne C, Dubrac S, Trieu-Cuot P, Dramsi S. Single Cell Stochastic Regulation of Pilus Phase Variation by an Attenuation-like Mechanism. Wessels MR, editor. PLoS Pathog. 2014 Jan 16;10(1):e1003860. doi:10.1371/journal.ppat.1003860

72. Danne C, Entenza JM, Mallet A, Briandet R, Débarbouillé M, Nato F, et al. Molecular Characterization of a Streptococcus gallolyticus Genomic Island Encoding a Pilus Involved in Endocarditis. The Journal of Infectious Diseases. 2011 Dec;204(12):1960–70. doi:10.1093/infdis/jir666

73. Martins M, Porrini C, Du Merle L, Danne C, Robbe-Masselot C, Trieu-Cuot P, et al. The Pil3 pilus of *Streptococcus gallolyticus* binds to intestinal mucins and to fibrinogen. Gut Microbes. 2016 Nov;7(6):526–32. doi:10.1080/19490976.2016.1239677

74. Martins M, Aymeric L, Du Merle L, Danne C, Robbe-Masselot C, Trieu-Cuot P, et al. *Streptococcus gallolyticus* Pil3 Pilus Is Required for Adhesion to Colonic Mucus and for Colonization of Mouse Distal Colon. J Infect Dis. 2015 Nov 15;212(10):1646–55. doi:10.1093/infdis/jiv307

75. Martins M, Du Merle L, Trieu-Cuot P, Dramsi S. Heterogeneous expression of Pil3 pilus is critical for Streptococcus gallolyticus translocation across polarized colonic epithelial monolayers. Microbes and Infection. 2020 Jan;22(1):55–9. doi:10.1016/j.micinf.2019.12.001

76. Jia D, Chen S. Adhesin RadD: the secret weapon of *Fusobacterium nucleatum*. Gut Microbes. 2024 Dec 31;16(1):2426617. doi:10.1080/19490976.2024.2426617

77. Kaplan CW, Lux R, Haake SK, Shi W. The *Fusobacterium nucleatum* outer membrane protein RadD is an arginine‐inhibitable adhesin required for inter‐species adherence and the structured architecture of multispecies biofilm. Molecular Microbiology. 2009 Jan;71(1):35–47. doi:10.1111/j.1365-2958.2008.06503.x

78. Kaplan CW, Ma X, Paranjpe A, Jewett A, Lux R, Kinder-Haake S, et al. *Fusobacterium nucleatum* Outer Membrane Proteins Fap2 and RadD Induce Cell Death in Human Lymphocytes. Infect Immun. 2010 Nov;78(11):4773–8. doi:10.1128/IAI.00567-10

79. Wang Z, Li B, Bao L, Chen Y, Yang J, Xu F, et al. Fusobacterium Nucleatum Aggravates Intestinal Barrier Impairment and Colitis Through IL-8 Induced Neutrophil Chemotaxis by Activating Epithelial Cells. JIR. 2024 Nov;Volume 17:8407–20. doi:10.2147/JIR.S470376

80. Xie H, Li Y, Fang Q, Xie H, Huang J, Wu Z, et al. SodA promotes immune evasion of *Streptococcus suis* by suppressing ROS accumulation and GSDMD-mediated mitochondrial disruption in neutrophils. Atack JM, editor. Microbiol Spectr. 2026 Jan 6;14(1):e01901-25. doi:10.1128/spectrum.01901-25

81. Purdy D, Cawthraw S, Dickinson JH, Newell DG, Park SF. Generation of a Superoxide Dismutase (SOD)-Deficient Mutant of *Campylobacter coli* : Evidence for the Significance of SOD in Campylobacter Survival and Colonization. Appl Environ Microbiol. 1999 Jun;65(6):2540–6. doi:10.1128/AEM.65.6.2540-2546.1999

82. Nusrat A, Von Eichel-Streiber C, Turner JR, Verkade P, Madara JL, Parkos CA. *Clostridium difficile* Toxins Disrupt Epithelial Barrier Function by Altering Membrane Microdomain Localization of Tight Junction Proteins. Barbieri JT, editor. Infect Immun. 2001 Mar;69(3):1329–36. doi:10.1128/IAI.69.3.1329-1336.2001

83. Von Eichel-Streiber C, Sauerborn M. Clostridium difficile toxin A carries a C-terminal repetitive structure homologous to the carbohydrate binding region of streptococcal glycosyltransferases. Gene. 1990 Jan;96(1):107–13. doi:10.1016/0378-1119(90)90348-U

84. Pothoulakis C, Sullivan R, Melnick DA, Triadafilopoulos G, Gadenne AS, Meshulam T, et al. Clostridium difficile toxin A stimulates intracellular calcium release and chemotactic response in human granulocytes. J Clin Invest. 1988 Jun 1;81(6):1741–5. doi:10.1172/JCI113514

85. Chen S, Sun C, Wang H, Wang J. The Role of Rho GTPases in Toxicity of Clostridium difficile Toxins. Toxins. 2015 Dec 2;7(12):5254–67. doi:10.3390/toxins7124874

86. Xu Q, Li Y, Zheng Y, Chen Y, Xu X, Wang M. Clostridium difficile toxin B-induced colonic inflammation is mediated by the FOXO3/PPM1B pathway in fetal human colon epithelial cells. Am J Transl Res. 2020;12(10):6204–19. PubMed PMID: 33194024; PubMed Central PMCID: PMC7653611.

87. Fletcher JR, Pike CM, Parsons RJ, Rivera AJ, Foley MH, McLaren MR, et al. Clostridioides difficile exploits toxin-mediated inflammation to alter the host nutritional landscape and exclude competitors from the gut microbiota. Nat Commun. 2021 Jan 19;12(1):462. doi:10.1038/s41467-020-20746-4

88. Yadav M, Zhang J, Fischer H, Huang W, Lutay N, Cirl C, et al. Inhibition of TIR Domain Signaling by TcpC: MyD88-Dependent and Independent Effects on Escherichia coli Virulence. Schneider DS, editor. PLoS Pathog. 2010 Sep 23;6(9):e1001120. doi:10.1371/journal.ppat.1001120

89. Gibold L, Garenaux E, Dalmasso G, Gallucci C, Cia D, Mottet-Auselo B, et al. The Vat-AIEC protease promotes crossing of the intestinal mucus layer by Crohn's disease-associated *Escherichia coli*: Vat-AIEC Favours Mucus Layer's Crossing by LF82 E. coli. Cellular Microbiology. 2016 May;18(5):617–31. doi:10.1111/cmi.12539

90. Lee A, White N, Van Der Walle CF. The intestinal zonula occludens toxin (ZOT) receptor recognises non‐native ZOT conformers and localises to the intercellular contacts. FEBS Letters. 2003 Dec 18;555(3):638–42. doi:10.1016/S0014-5793(03)01348-6

91. Mahendran V, Liu F, Riordan SM, Grimm MC, Tanaka MM, Zhang L. Examination of the effects of Campylobacter concisus zonula occludens toxin on intestinal epithelial cells and macrophages. Gut Pathog. 2016 Dec;8(1):18. doi:10.1186/s13099-016-0101-9

92. Fasano A, Baudry B, Pumplin DW, Wasserman SS, Tall BD, Ketley JM, et al. Vibrio cholerae produces a second enterotoxin, which affects intestinal tight junctions. Proc Natl Acad Sci USA. 1991 Jun 15;88(12):5242–6. doi:10.1073/pnas.88.12.5242

93. Nakagawa I, Amano A, Kuboniwa M, Nakamura T, Kawabata S, Hamada S. Functional Differences among FimA Variants of *Porphyromonas gingivalis* and Their Effects on Adhesion to and Invasion of Human Epithelial Cells. Infect Immun. 2002 Jan;70(1):277–85. doi:10.1128/IAI.70.1.277-285.2002

94. Mishra A, Wu C, Yang J, Cisar JO, Das A, Ton‐That H. The *Actinomyces oris* type 2 fimbrial shaft FimA mediates co‐aggregation with oral streptococci, adherence to red blood cells and biofilm development. Molecular Microbiology. 2010 Aug;77(4):841–54. doi:10.1111/j.1365-2958.2010.07252.x

95. Cai J, Chen J, Guo H, Pan Y, Zhang Y, Zhao W, et al. Recombinant fimbriae protein of Porphyromonas gingivalis induces an inflammatory response via the TLR4/NF-κB signaling pathway in human peripheral blood mononuclear cells. Int J Mol Med. 2019 Jan 21. doi:10.3892/ijmm.2019.4069

96. Bagaitkar J, Demuth DR, Daep CA, Renaud DE, Pierce DL, Scott DA. Tobacco Upregulates P. gingivalis Fimbrial Proteins Which Induce TLR2 Hyposensitivity. Ratner AJ, editor. PLoS ONE. 2010 May 4;5(5):e9323. doi:10.1371/journal.pone.0009323

97. Katz J, Yang QB, Zhang P, Potempa J, Travis J, Michalek SM, et al. Hydrolysis of Epithelial Junctional Proteins by *Porphyromonas gingivalis* Gingipains. Infect Immun. 2002 May;70(5):2512–8. doi:10.1128/IAI.70.5.2512-2518.2002

98. Tada H, Sugawara S, Nemoto E, Takahashi N, Imamura T, Potempa J, et al. Proteolysis of CD14 on Human Gingival Fibroblasts by Arginine-Specific Cysteine Proteinases from *Porphyromonas gingivalis* Leading to Down-Regulation of Lipopolysaccharide-Induced Interleukin-8 Production. Infect Immun. 2002 Jun;70(6):3304–7. doi:10.1128/IAI.70.6.3304-3307.2002

99. Ciaston I, Budziaszek J, Satala D, Potempa B, Fuchs A, Rapala-Kozik M, et al. Proteolytic Activity-Independent Activation of the Immune Response by Gingipains from Porphyromonas gingivalis. Goldberg JB, editor. mBio. 2022 Jun 28;13(3):e03787-21. doi:10.1128/mbio.03787-21

100. Chen T, Nakayama K, Belliveau L, Duncan MJ. *Porphyromonas gingivalis* Gingipains and Adhesion to Epithelial Cells. Clements JD, editor. Infect Immun. 2001 May;69(5):3048–56. doi:10.1128/IAI.69.5.3048-3056.2001

101. Dashper SG, Cross KJ, Slakeski N, Lissel P, Aulakh P, Moore C, et al. Hemoglobin hydrolysis and heme acquisition by *Porphyromonas gingivalis*. Oral Microbiology and Immunology. 2004 Feb;19(1):50–6. doi:10.1046/j.0902-0055.2003.00113.x

102. Lee JY, Miller DP, Wu L, Casella CR, Hasegawa Y, Lamont RJ. Maturation of the Mfa1 Fimbriae in the Oral Pathogen Porphyromonas gingivalis. Front Cell Infect Microbiol. 2018 May 9;8:137. doi:10.3389/fcimb.2018.00137

103. Van Der Post S, Subramani DB, Bäckström M, Johansson MEV, Vester-Christensen MB, Mandel U, et al. Site-specific O-Glycosylation on the MUC2 Mucin Protein Inhibits Cleavage by the Porphyromonas gingivalis Secreted Cysteine Protease (RgpB). Journal of Biological Chemistry. 2013 May;288(20):14636–46. doi:10.1074/jbc.M113.459479

104. Takeuchi H, Sasaki N, Yamaga S, Kuboniwa M, Matsusaki M, Amano A. Porphyromonas gingivalis induces penetration of lipopolysaccharide and peptidoglycan through the gingival epithelium via degradation of junctional adhesion molecule 1. Darveau R, editor. PLoS Pathog. 2019 Nov 7;15(11):e1008124. doi:10.1371/journal.ppat.1008124

105. Tubero Euzebio Alves V, Alves T, Silva Rovai E, Hasturk H, Van Dyke T, Holzhausen M, et al. Arginine-specific gingipains (RgpA/RgpB) knockdown modulates neutrophil machinery. Journal of Oral Microbiology. 2024 Dec 31;16(1):2376462. doi:10.1080/20002297.2024.2376462

106. Lourbakos A, Potempa J, Travis J, D'Andrea MR, Andrade-Gordon P, Santulli R, et al. Arginine-Specific Protease from *Porphyromonas gingivalis* Activates Protease-Activated Receptors on Human Oral Epithelial Cells and Induces Interleukin-6 Secretion. Moore RN, editor. Infect Immun. 2001 Aug;69(8):5121–30. doi:10.1128/IAI.69.8.5121-5130.2001

107. Inaba H, Sugita H, Kuboniwa M, Iwai S, Hamada M, Noda T, et al. *Porphyromonas gingivalis* promotes invasion of oral squamous cell carcinoma through induction of proMMP9 and its activation: Promotion of oral cancer invasion by *P. gingivalis*. Cell Microbiol. 2014 Jan;16(1):131–45. doi:10.1111/cmi.12211

108. Into T, Inomata M, Kanno Y, Matsuyama T, Machigashira M, Izumi Y, et al. Arginine-specific gingipains from *Porphyromonas gingivalis* deprive protective functions of secretory leucocyte protease inhibitor in periodontal tissue. Clinical and Experimental Immunology. 2006 Jul 12;145(3):545–54. doi:10.1111/j.1365-2249.2006.03156.x

109. Zhou X, Gao J, Hunter N, Potempa J, Nguyen K. Sequence‐independent processing site of the C ‐terminal domain (CTD) influences maturation of the RgpB protease from *P orphyromonas gingivalis* . Molecular Microbiology. 2013 Sep;89(5):903–17. doi:10.1111/mmi.12319

110. Discipio RG, Daffern PJ, Kawahara M, Pike R, Travis J, Hugli TE. Cleavage of human complement component C5 by cysteine proteinases from *Porphyromonas (Bacteroides) gingivalis* . Prior oxidation of C5 augments proteinase digestion of C5. Immunology. 1996 Apr;87(4):660–7. doi:10.1046/j.1365-2567.1996.478594.x

111. Stathopoulou PG, Benakanakere MR, Galicia JC, Kinane DF. The host cytokine response to *Porphyromonas gingivalis* is modified by gingipains. Oral Microbiology and Immunology. 2009 Feb;24(1):11–7. doi:10.1111/j.1399-302X.2008.00467.x

112. O'Brien-Simpson NM, Pathirana RD, Walker GD, Reynolds EC. *Porphyromonas gingivalis* RgpA-Kgp Proteinase-Adhesin Complexes Penetrate Gingival Tissue and Induce Proinflammatory Cytokines or Apoptosis in a Concentration-Dependent Manner. Infect Immun. 2009 Mar;77(3):1246–61. doi:10.1128/IAI.01038-08

113. Ito R, Ishihara K, Shoji M, Nakayama K, Okuda K. Hemagglutinin/Adhesin domains of *Porphyromonas gingivalis* play key roles in coaggregation with *Treponema denticola*. FEMS Immunol Med Microbiol. 2010 Dec;60(3):251–60. doi:10.1111/j.1574-695X.2010.00737.x

114. Lewis JP, Dawson JA, Hannis JC, Muddiman D, Macrina FL. Hemoglobinase Activity of the Lysine Gingipain Protease (Kgp) of Porphyromonas gingivalis W83. J Bacteriol. 1999 Aug 15;181(16):4905–13. doi:10.1128/JB.181.16.4905-4913.1999
